# Characterisation of extraembryonic endoderm-like cells from mouse embryonic fibroblasts induced using chemicals alone

**DOI:** 10.1186/s13287-020-01664-0

**Published:** 2020-04-16

**Authors:** Xia He, Guangfan Chi, Meiying Li, Jinying Xu, Lihong Zhang, Yaolin Song, Lina Wang, Yulin Li

**Affiliations:** 1 The Key Laboratory of Pathobiology, Ministry of Education, Department of Pathology, College of Basic Medical Sciences, Jilin University, Changchun, 130021 Jilin People’s Republic of China; 2 Department of Pathology, The Affiliated Hospital of Qingdao University, Qingdao, 266000 Shandong People’s Republic of China; 3 Department of Paediatrics, The First Hospital of Jilin University, Changchun, 130021 Jilin People’s Republic of China

**Keywords:** Chemicals, ciXEN cells, Energy metabolism, Induced hepatocytes, Induced extraembryonic endoderm cells

## Abstract

**Background:**

The development of somatic reprogramming, especially purely chemical reprogramming, has significantly advanced biological research. And chemical-induced extraembryonic endoderm-like (ciXEN) cells have been confirmed to be an indispensable intermediate stage of chemical reprogramming. They resemble extraembryonic endoderm (XEN) cells in terms of transcriptome, reprogramming potential, and developmental ability in vivo. However, the other characteristics of ciXEN cells and the effects of chemicals and bFGF on the in vitro culture of ciXEN cells have not been systematically reported.

**Methods:**

Chemicals and bFGF in combination with Matrigel were used to induce the generation of ciXEN cells derived from mouse embryonic fibroblasts (MEFs). RNA sequencing was utilised to examine the transcriptome of ciXEN cells, and PCR/qPCR assays were performed to evaluate the mRNA levels of the genes involved in this study. Hepatic functions were investigated by periodic acid-Schiff staining and indocyanine green assay. Lactate production, ATP detection, and extracellular metabolic flux analysis were used to analyse the energy metabolism of ciXEN cells.

**Results:**

ciXEN cells expressed XEN-related genes, exhibited high proliferative capacity, had the ability to differentiate into visceral endoderm in vitro, and possessed the plasticity allowing for their differentiation into induced hepatocytes (iHeps). Additionally, the upregulated biological processes of ciXEN cells compared to those in MEFs focused on metabolism, but their energy production was independent of glycolysis. Furthermore, without the cocktail of chemicals and bFGF, which are indispensable for the generation of ciXEN cells, induced XEN (iXEN) cells remained the expression of XEN markers, the high proliferative capacity, and the plasticity to differentiate into iHeps in vitro.

**Conclusions:**

ciXEN cells had high plasticity, and energy metabolism was reconstructed during chemical reprogramming, but it did not change from aerobic oxidation to glycolysis. And the cocktail of chemicals and bFGF were non-essential for the in vitro culture of ciXEN cells.

## Background

The emergence of transcription factor (TF)-mediated induced pluripotent stem cells (iPSCs) [1, 2], which reverse developmental programmes, has attracted great attention in the scientific community and has created new opportunities for cell replacement therapies and regenerative medicine. However, somatic reprogramming is a process of complex and progressive cell fate conversion. And the intermediate-stage cells, which maintain plasticity to differentiate into several functional cellular types, are crucial to this process. Nevertheless, the study of this stage cells induced by TFs has been challenging, as they are diverse and led to a lack of consistent reporting. TF-mediated reprogramming requires cells to go through a primitive streak-like state [3], while reprogramming of canine fibroblasts by genetic manipulation has been found to yield XEN-like cells that exhibit endodermal plasticity [4]. However, other study has found that XEN-like cells and iPSCs were induced concomitantly, but independently during reprogramming [5]. More recently, the generation of chemical-induced pluripotent stem cells (ciPSCs) from mouse [6–8] and goat [9] solely through the use of chemicals has been successful. And previous studies have confirmed that ciXEN cells represent an indispensable intermediate cell state during chemical-based multipotential reprogramming [10]. This provides an idealised model for the systematical investigation of ciXEN cells.

And it has been reported that the gene expression profiles of ciXEN cells resemble those of XEN cells [10], which originate from primitive endoderm (PrE). And studies have shown that ciXEN cells differentiated into parietal endoderm (PE) in the chimeric assays, and even were transformed into hepatocytes [11], which is supported by the fact that XEN cells contribute to the definitive endoderm (DE) [12]. Additionally, it has been shown that chemicals and bFGF, which are required for the long-term culture of ciXEN cells in vitro, are also suitable for the maintenance of XEN cells [10]. However, the in vitro culture of XEN cells does not require these chemicals and bFGF, and no studies have thus far reported the characteristics of ciXEN cells after removing chemicals and bFGF.

In addition, multipotential reprogramming is accompanied by changes in metabolism as well as in gene expression patterns and cellular morphology [13–15]. Recent studies have found that during the early stage of somatic reprogramming, the expression of metabolism-related genes changes significantly [16, 17]. With respect to extraembryonic cell lineages, trophoblast cells have been shown to depend on aerobic respiration for energy [18], while the metabolic patterns of XEN cells are relatively complex and there are no consistent reports in existence. F9 cells between the early passage and late passage demonstrated the reverse metabolic shift during differentiation into XEN cells [19]. Further, ciXEN cells have only been investigated at an intermediate stage of chemical reprogramming, and it is unknown how their metabolic patterns changed. Based on these researches described above, we propose the following hypothesis: the cocktail of chemicals and bFGF can successfully reprogramme fibroblasts into ciXEN cells, and this process may be accompanied by metabolic remodelling; in addition, after the generation of ciXEN cells, this cocktail may be non-essential for maintaining the in vitro characteristics of ciXEN cells.

Consequently, the current study elucidated the characteristics of ciXEN cells and their metabolic patterns and investigated the effects of small molecules and bFGF on the long-term culture of ciXEN cells in vitro.

## Methods

### Cell culture

MEFs were derived from CF-1 mice embryos as described in our previous study [20]. Mouse neonatal fibroblasts (MNFs) were isolated from the dermal skins of neonatal mice. After being spliced into small pieces, the dermis was digested overnight with 0.2% Dispase (Gibco, NY, USA) at 4 °C, then incubated with collagenase I (Gibco) at 37 °C, and finally collected cells with centrifugation. MEFs and MNFs were both cultured in fibroblast culture medium (FCM) containing high glycose DMEM (Hyclone, UT, USA), 10% foetal bovine serum (FBS), 2 mM GlutaMAX™, 100 U/ml penicillin, and 100 μg/ml streptomycin (all from Gibco) at 37 °C in a 5% CO_2_ atmosphere.

### Generation of ciXEN cells

For obtaining ciXEN cells, MEFs and MNFs at passage 2 or 3 were seeded into a 6-well plate pre-coated with Matrigel (Corning, MA, USA) at a density of 3 × 10^4^ cells, 4 × 10^4^ cells, or 5 × 10^4^ cells per well. After 24 h, the FCM was changed to the induced medium (IM) (KnockOut DMEM supplemented with 10% knockout serum replacement (both from Gibco), 10% FBS, 100 ng/ml bFGF, 0.5 mM VPA (Sigma, USA), 20 μM CHIR99021, 10 μM RepSox, 5 μM Parnate, 50 μM forskolin (all from MCE, China), 0.05 μM AM580, and 5 μM EPZ004777 (both from Tocris, USA)), which resembled Deng’s recipe [10]. The medium was replaced every 4 days. After 16 days, cell clones were mechanically harvested. Once the epithelial-like cells crawled out of the selected clones, the concentrations of bFGF, CHIR99021, and forskolin in IM were reduced to 25 ng/ml, 10 μM, and 10 μM, respectively, according to previous studies [10]. This medium, which is suitable for long-term culture of ciXEN cells, is designated as the maintenance medium (MM).

To verify whether a metabolic shift occurred during the generation of ciXEN cells, we additionally added PS48 (an activator of PDK1 which promotes glycolysis, MCE; 5 μM) to the IM. Other procedures were the same as the chemical-induced process.

### Spontaneous differentiation of ciXEN cells and obtaining iXEN cells

ciXEN cells were resuspended in FCM and performed hanging-drop culture at a density of 5 × 10^5^ cells per drop. The medium was changed every 4 days. After 8 days, the cell masses were transferred to a Matrigel-coated 12-well plate and then conducted subsequent experiment.

To gain iXEN cells, we reseeded ciXEN cells into the Matrigel-coated 6-well plate and replaced the MM with high glycose DMEM supplemented with 1% FBS or 10% FBS.

### Generation of visceral endoderm (VE) from ciXEN cells

ciXEN cells were replated into Matrigel-coated culture plates. Fresh medium containing high glucose DMEM, 1× B27, 1× N2, 2 mM GlutaMAX, 1% non-essential amino acids (NEAA) (all from Gibco), and 50 ng/ml BMP4 was used to replace MM and was changed every other day. This induction process lasted 7 days.

### Induction of iHeps

For hepatic differentiation, ciXEN cells were reseeded into Matrigel-coated 6-well plates. The induction process was divided into two stages: In stage I, ciXEN cells were cultured in the hepatic induced medium I (HIM I) containing high glucose DMEM, 1% N2, 1% B27, 1% ITS-X (Gibco), 2 mM GlutaMAX™, 0.1 mM NEAA, 20 ng/ml HGF, 20 ng/ml EGF, 10 ng/ml BMP4, 10 ng/ml bFGF, and 20 ng/ml Activin A for 9 days. In stage II, they were then cultured in hepatic induced medium II (HIM II), which was HIM I supplemented with 10 ng/ml OsM and 1 μM dexamethasone but withdrawing Activin A (all cytokines from PeproTech, USA) for additional 10 days. This medium was changed every 3 days.

Generation of iHeps from induced XEN cells was performed using the same induction protocol.

### PCR/qPCR and RNA sequencing

#### PCR/qPCR

Total RNA was extracted using a RNeasy Plus Mini Kit (Qiagen, Germany) according to the manufacturer’s instructions, and then, 1 μg RNA was converted into first-strand cDNA using the TransScript First-Strand cDNA Synthesis SuperMix (TransGen Biotech, China). PCR/qPCR was carried out according to our previous protocol [21]. The relative expression was calculated with the comparative Ct method. The primer sequences are included in Table S1 (Additional file 2).

#### RNA sequencing

One to 2 μg total RNA with rRNA removed was used to build sequencing libraries using a KAPA Stranded RNA-Sequence Library Prep Kit (Illumina, California). RNA sequencing was performed using an Illumina Hiseq 4000 Sequenator. Original RNA sequence data were uploaded to the Gene Expression Omnibus database: GSE136824; https://www.ncbi.nlm.nih.gov/geo/query/acc.cgi?acc=GSE136824. We conducted a cluster analysis of differentially expressed genes (DEGs) by fragment per kilobase of transcript per million mapped (FPKM) reads, generated a volcano plot of DEGs, and performed principal component analysis (PCA) using genes with significantly different geometric means in all samples (*p* value < 0.05). Additionally, Gene Ontology (GO) analysis, Venn diagram, and Kyoto Encyclopaedia of Genes and Genomes (KEGG) pathway analysis were summarised using custom programs, including Python (version 2.7), R (version 3.5.0), and Shell (*p* ≤ 0.05).

### Immunofluorescence staining

Cells were fixed with 4% paraformaldehyde (PFA; Ding Guo, China) for 15 min, permeabilized with 0.1% Triton X-100 (Sigma) for 15 min, blocked with 1% BSA for 30 min at room temperature, and then incubated overnight with primary antibody targeting Vimentin, Desmin, Nestin, Nanog, Foxa2, Gata4, E-cadherin, Oct4, Hnf4a (Cell Signalling Technology, MA, USA), Foxa3 (Atlas Antibodies, China), Sox2 (GeneTex, USA), Asgpr1 (Bioscience, USA), Afp, and Sox17 (R&D Systems, UK) at 4 °C. The following day, cells were incubated with Alexa Fluor® 488/555-conjugated goat anti-mouse/rabbit antibody (Cell Signalling Technology) or Alexa Fluor® 488-conjugated donkey anti-goat antibody (Absin Bioscience, China) for 1 h in the dark. Nuclei were stained with 10 μg/ml Hoechst33342 (Invitrogen, USA). Cells were visualised by fluorescence microscope (Olympus IX71, Tokyo, Japan).

### Western blot

Cells were lysed in buffer containing RIPA and PSMF (TransGen Biotech, China). Proteins were quantified using a BCA protein assay kit (Beyotime Biotechnology) according to the manufacturer’s specifications. After denaturation, proteins were separated on 10% polyacrylamide gels and then transfered to polyvinylidene fluoride (PVDF) membranes (Millipore, CA, USA). After being blocked, the membranes were incubated overnight with primary antibodies for E-cadherin, Vimentin, Gata4, Sox2, Foxa2, Desmin (Cell Signalling Technology), Sox17 (R&D Systems), and Gadph (ProteinTech) at 4 °C and then with secondary antibodies on the next day. Finally, the protein bands were visualised with hypersensitive chemiluminescence (Beyotime Biotechnology).

### EdU assay and cell cycle assay

To analyse the proliferative capacity, 2 × 10^3^ cells were seeded into 96-well plates and then incubated with the medium containing 50 μM EdU for 2 h. Subsequently, the EdU assay was performed using a Cell-Light™ EdU Apollo® 555 Kit (RiboBio, China) according to the manufacturer’s instructions. Cells were examined by fluorescence microscope. The positive rate was analysed using the ImageJ software.

To detect the cell cycle, we collected cells by centrifugation and they were fixed overnight with ice-cold 75% ethyl alcohol at 4 °C. The following day, cells were resuspended with propidium iodide/RNase (Cell Signalling Technology) and incubated for 15 min. Finally, the cell cycle was examined by flow cytometry (FACS Calibur flow cytometer; BD Biosciences, USA). The cell proliferation index (PI) was calculated as follows: PI = (*S* + *G*2/*M*)/(*G*0/*G*1 + *S* + *G*2/*M*) × 100%.

### Karyotyping analysis

The karyotyping analysis of ciXEN cells at different passages and MEFs in the logarithmic growth phase was performed as previously reported [22].

### Transmission electron microscopy (TEM)

Cells collected by centrifugation were fixed with 4% glutaraldehyde (Sigma) and then refixed by 1% osmium tetroxide (Sigma) for 2 h. Next, cell aggregates underwent gradient dehydration, pre-soaking, and polymerisation and were finally cut into ultrathin sections in order to perform dioxyuranium acetate staining and lead citrate staining. The thin sections were observed with TEM (FEI Tecnai G2; America).

### Alkaline phosphatase (ALP)

ALP activity was detected by a ALP assay kit (Solarbio, China) in accordance with the manufacturer’s protocol. MEFs and ciXEN cells were fixed with 4% PFA for 15 min, incubated with pre-mixed ALP incubated buffer for 15 min, and then redyed with nuclear fast red. Finally, the images were captured by an inverted microscope (Olympus IX71).

### Hepatic function assays

#### Periodic acid-Schiff (PAS) staining

MEFs, iHeps, and ciXEN cells were fixed with 4% PFA and washed three times with double distilled water (ddH_2_O). Then, these cells were stained with a PAS kit (Sigma) according to the manufacturer’s instructions.

#### Indocyanine green (ICG) assay

iHeps and ciXEN cells were cultured in the medium supplemented with 1 mg/ml ICG (Sigma) for 2 h at 37 °C. We then added fresh medium without ICG into the culture dishes and continued to incubate overnight at 37 °C. The cells were observed by an inverted microscope.

### Lactate production

The lactate levels of MEFs and ciXEN cells were detected using a lactate content detection kit (Solarbio, China). We replated these cells on 6 cm culture dishes for 3 days and then detected the lactate production according to the manufacturer’s instructions. Finally, lactate concentrations were calculated from cell numbers.

### Extracellular metabolic flux analysis

We seeded MEFs and ciXEN cells onto Matrigel-coated XF96 well plates (Seahorse Bioscience, USA). To obtain extracellular acidification rate (ECAR), the cells were metabolically perturbed by glucose (10 mM), oligomycin (1 mM), and 2-deoxyglucose (50 mM) (all from Sigma). For mitochondria stress tests, oxygen consumption rate (OCR) was measured by supplementing with DMEM, 1 μM oligomycin, 2 μM FCCP, and 1 μM rotenone and 1 μM antimycin A (all from Sigma). The final measurements were normalised by the number of viable cells.

### Adenosine triphosphate (ATP) detection

ciXEN cells and MEFs were replated on Matrigel-coated 6-well plates and lysed with 200 μl ATP detection cracking solution (Beyotime Biotechnology). Then, the lysate was used for the following experiments according to the manufacturer’s protocol. The final production of ATP was calculated from the protein concentration of ciXEN cells and MEFs.

### Statistical analysis

Results are presented as means ± SEM. The statistical graphs were carried out using GraphPad Prism 5 (GraphPad software). We applied two-way ANOVA among mutiple groups and *t* tests between two groups to calculate statistical significance. Each experiment was repeated at least three times. *p* < 0.05 was considered statistically significant.

## Results

### Reprogramming of ciXEN cells from MEFs induced by chemicals

**Fig. 1:**
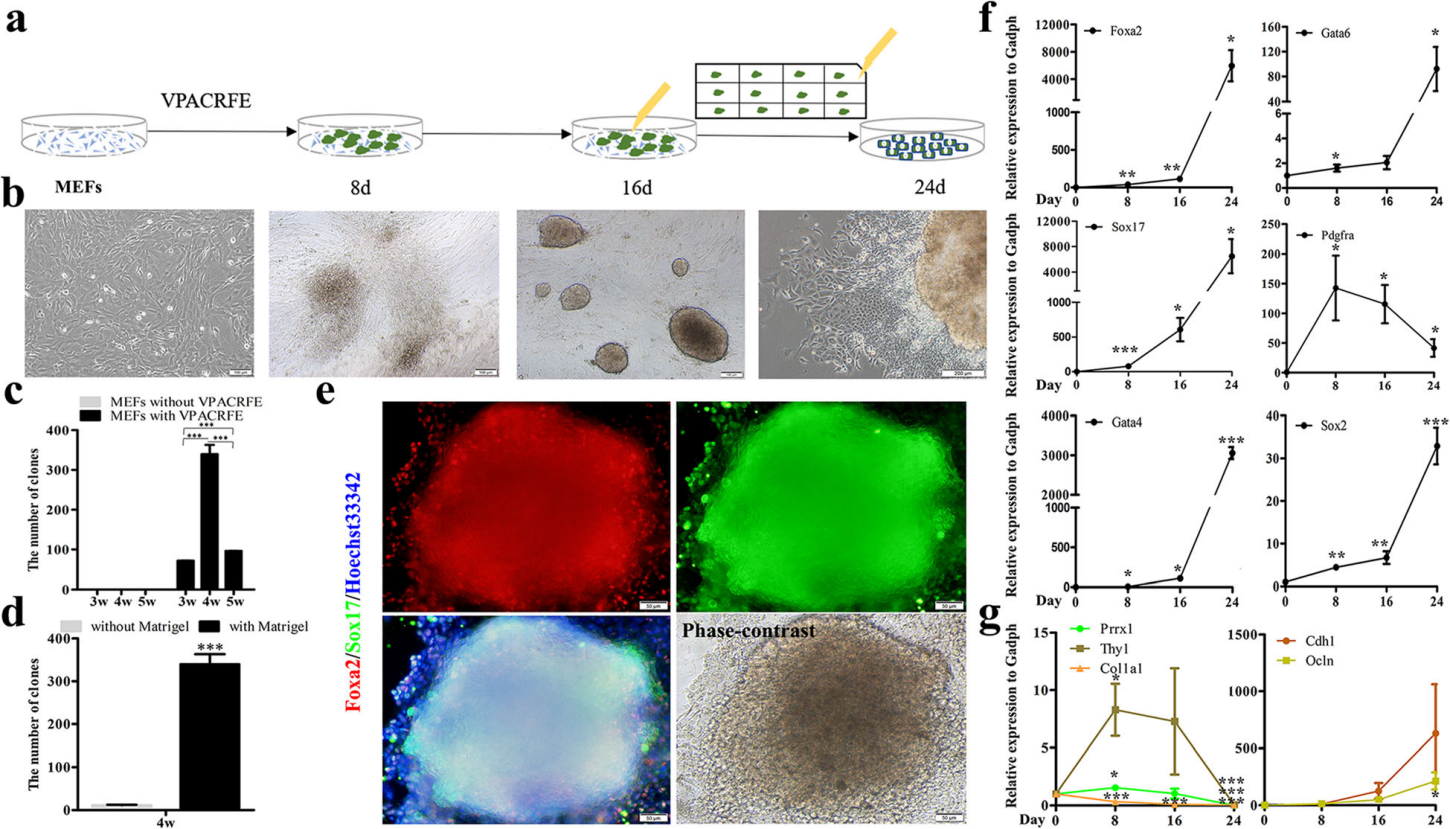
Generation of ciXEN cells. **a** Schematic diagram for obtaining ciXEN cells from MEFs using small molecules. **b** Morphological appearances of MEFs, cell colonies (treatment with chemicals for 8 days (8d) and 16 days (16d)) (bar, 100 μm), and epithelial-like cells emerging out of the selected clone (24d) (bar, 200 μm). **c** Numbers of cell clones with different numbers of starting cells: 3w, 4w, and 5w. **d** Effect of Matrigel on the formation of clones from MEFs with 4w initial cells. **e** Co-immunostaining (Foxa2 and Sox17) and phase-contrast image of cell clone (bar, 50 μm). qPCR results for the expression of XEN-related genes and *Sox2* (**f**), and fibroblast markers and epithelial markers (**g**) from day 0 to day 24. V, VPA; P, Parnate; A, AM580; C, CHIR99021; R, RepSOX; F, forskolin; E, EPZ004777; 3w, 3 × 10^4^ cells; 4w, 4 × 10^4^ cells; 5w, 5 × 10^4^ cells. *p < 0.05, **p < 0.01, ***p < 0.001. Mean ± SEM, *t* test and two-way ANOVA, *n* ≥ 3

To generate ciXEN cells from MEFs, an IM containing chemical cocktail and bFGF—originally reported to induce chemical reprogramming without any TFs [10]—was used in our study (Fig. 1a). The number of cell clones as determined by manual count was used as a readout to estimate the reprogramming efficiency. After determining the optimal clone formation rate with different number of starting cells, we selected a density 4 × 10^4^ cells per well in six-well plates for subsequent experiments (Fig. 1c). As early as day 6, distinct cell aggregates could be observed. After continual chemical cocktail administration for 16 days, most cell aggregates had a clear boundary. We then handpicked clones and transferred them into a 12-well plate pre-coated with Matrigel (one clone per well). Approximately 1 week later, numerous epithelial-like cells emerged out of the clones (Fig. 1a, b). And our results demonstrated that the efficiency of the cells grown on the plates pre-coated with Matrigel was 31.47 ± 3.86 times greater than that of those grown on the Matrigel-uncoated plates (Fig. 1d). This finding reveals that Matrigel in combination with chemicals and bFGF facilitated the generation of cell clones from MEFs.

And we then found that these cell clones at day 16 co-expressed *Foxa2* and *Sox17* (Fig. 1e). Furthermore, a crucial point in successful reprogramming is to gain the properties of the desired cells and eliminate the characteristics of the original cells. Our results reveal that the mRNA levels of XEN markers (*Gata6*, *Foxa2*, *Sox17*, and *Gata4*) were significantly upregulated during this period. And *Pdgfra*, another XEN gene, showed an upward trend during the first 8 days and then decreased from day 8 to day 24 but still be detected. Additionally, *Sox2* and *Epcam* increased significantly from day 0 to day 24 (Fig. 1f and Additional file 1: Figure S1a). Simultaneously, the mRNA levels of the fibroblast markers (*Thy1* and *Prrx1*) increased from day 0 to day 8 before decreasing from day 8 to day 24, with *Thy1* particularly showing a significant decrease. And the level of *Col1a1* decreased continually throughout the experiment (Fig. 1g). *Cxcr4*, a marker of DE, increased gradually (Additional file 1: Figure S1b). Additionally, qPCR analysis showed that the levels of epithelial markers (*Cdh1* and *Ocln*) increased, particularly from day 16 to day 24 (Fig. 1g), while the mRNA levels of the mesenchymal markers (*Zeb1*, *Vimentin*, and *Twist2*) were downregulated. *Snai1*, another mesenchymal gene, continually maintained an upward trend (Additional file 1: Figure S1c). Studies have shown that *Snai1* is a marker of the parietal endoderm (PE) [23]. And the protein levels of *E-cadherin* and *Vimentin* were consistent with their mRNA levels (Additional file 1: Figure S1d). These results indicate that a mesenchymal epithelial transition (MET) occurred during this chemical induction process.

This chemical recipe used for MEF reprogramming was also used to treat MNFs. We found that cells in the chemically induced clones were loosely arranged (Additional file 1: Figure S1e), which also occurred in some MEF-derived clones. Besides that, the highest number of clones was obtained using an initial cell number of 3 × 10^4^ (Additional file 1: Figure S1f), and these clones co-expressed *Sox17* and *Foxa2* (Additional file 1: Figure S1g). These results indicated that the chemical cocktail was suitable not only for the reprogramming of MEFs, but also for that of MNFs.

### Characteristics of ciXEN cells

**Fig. 2:**
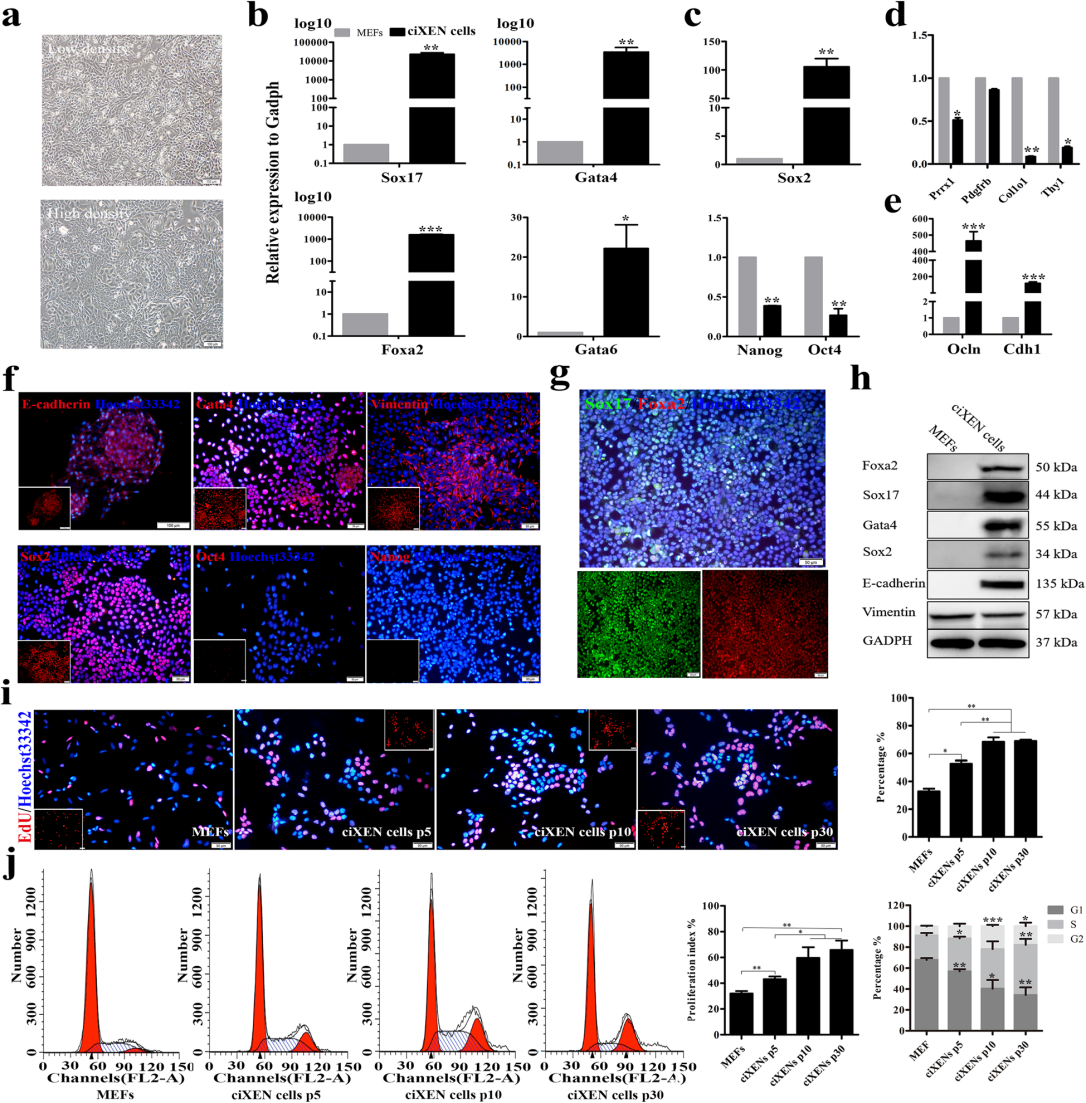
Characteristics of ciXEN cells at passage 5. Morphological appearances of ciXEN cells at low density and high density (bar, 100 μm) (**a**). qPCR results for the expression of XEN-related genes (*Foxa2*, *Sox17*, *Gata4*, and *Gata6*) (**b**), pluripotency markers (*Sox2*, *Oct4*, and *Nanog*) (**c**), fibroblast markers (*Pdgfrb*, *Prrx1*, *Thy1*, and *Col1a1*) (**d**), and epithelial-related genes (**e**). **f**, **g** Immunofluorescence of Sox2, Gata4, E-cadherin, vimentin, Oct4, Nanog, Sox17, and Foxa2 in ciXEN cells (bar, 50 μm). **h** Western blot analysis for the expression of Foxa2, Sox17, Gata4, Sox2, E-cadherin, and vimentin in MEFs and ciXEN cells. **i** EdU assay for proliferative ability of MEFs and ciXEN cells at p5, p10, and p30 (bar, 50 μm). **j** Detection of cell cycle by flow cytometry. The percentage of G1-, S-, and G2-phase in the cell cycle and the PI of MEFs and ciXEN cells at p5, p10, and p30. p5, passage 5; p10, passage 10; p30, passage 30. *p < 0.05, **p < 0.01, ***p < 0.001. Mean ± SEM, *t* test and two-way ANOVA, *n* = 3

Subsequentially, we detected the characteristics of ciXEN cells derived from the selected clones. ciXEN cells had two distinct morphological characteristics: dispersed cells at low density and epithelioid cells at high density (Fig. 2a) that resembled XEN cells from mouse blastocysts [24]. Compared to that in MEFs, the mRNA levels of XEN markers in ciXEN cells at passage 5 significantly increased (Fig. 2b). In addition to *Epcam* and *Pdgfra*, they also expressed the endoderm gene, *Cxcr4* (Additional file 1: Figure S2a and S2b). Interestingly, these cells also demonstrated high *Sox2* expression, consistent with immunostaining. Nevertheless, we could not detect pluripotent genes at either the mRNA or protein level (Fig. 2c, f), indicating that the ciXEN cells had not yet reached the pluripotent stage. In addition, because the ciXEN cells did not express *Oct4*, we identified them as non-XEN progenitor cells [25]. And the mRNA levels of fibroblast-related genes in ciXEN cells were significantly reduced compared to those in MEFs, while mRNA levels of *Ocln* and *Cdh1* were significantly higher than those in MEFs (Fig. 2d, e). Additionally, they positively expressed *E-cadherin*, *Gata4*, and *Vimentin* (Fig. 2f), which indicates that the transformation of MEFs into ciXEN cells was incomplete. To determine the purity of ciXEN cells, co-immunostaining was used. Our result reveals that the percentage of cells expressing *Sox17* and *Foxa2* approached 100% (Fig. 2g). These results were also confirmed by our western blot analysis (Fig. 2h).

Additionally, the proliferative ability of ciXEN cells mostly in the S phase of the cell cycle was significantly higher than that of MEFs, and most stable between passage 10 and passage 30, with proliferative ability increased relative to passage 5 (Fig. 2i, j). To further distinguish between MEFs and ciXEN cells, TEM was used to study their ultrastructure. Our results suggest that pseudopodia were present over the surface of MEFs, the chromatin was arranged compactly, and the cytoplasm contained endoplasmic reticulum and mitochondria. In case of ciXEN cells, we observed a smooth cell surface with a unique cilium-like structure and loosely arranged chromatin; endoplasmic reticulum and mitochondria were also observed, but mitochondria were circular instead of elongated (Additional file 1: Figure S2c).

Furthermore, these cells retained epithelioid morphologies through a series of passages, and their karyotype remained unaltered (Additional file 1: Figure S2d and S2e). The expression levels of epithelial markers and XEN-related genes remained high throughout, while the mRNA levels of fibroblast-related genes were nearly impossible to detect (Additional file 1: Figure S2f and S2g). Interestingly, during continuous subculturing, the expression of *Sox2* was downregulated, except at passage 30, and the expression of pluripotency genes was not detected (Additional file 1: Figure S2h). These results indicate that ciXEN cells maintained their characteristics during expansion in vitro, an important condition for the practical applications.

### In-depth transcriptomic analyses of ciXEN cells

**Fig. 3:**
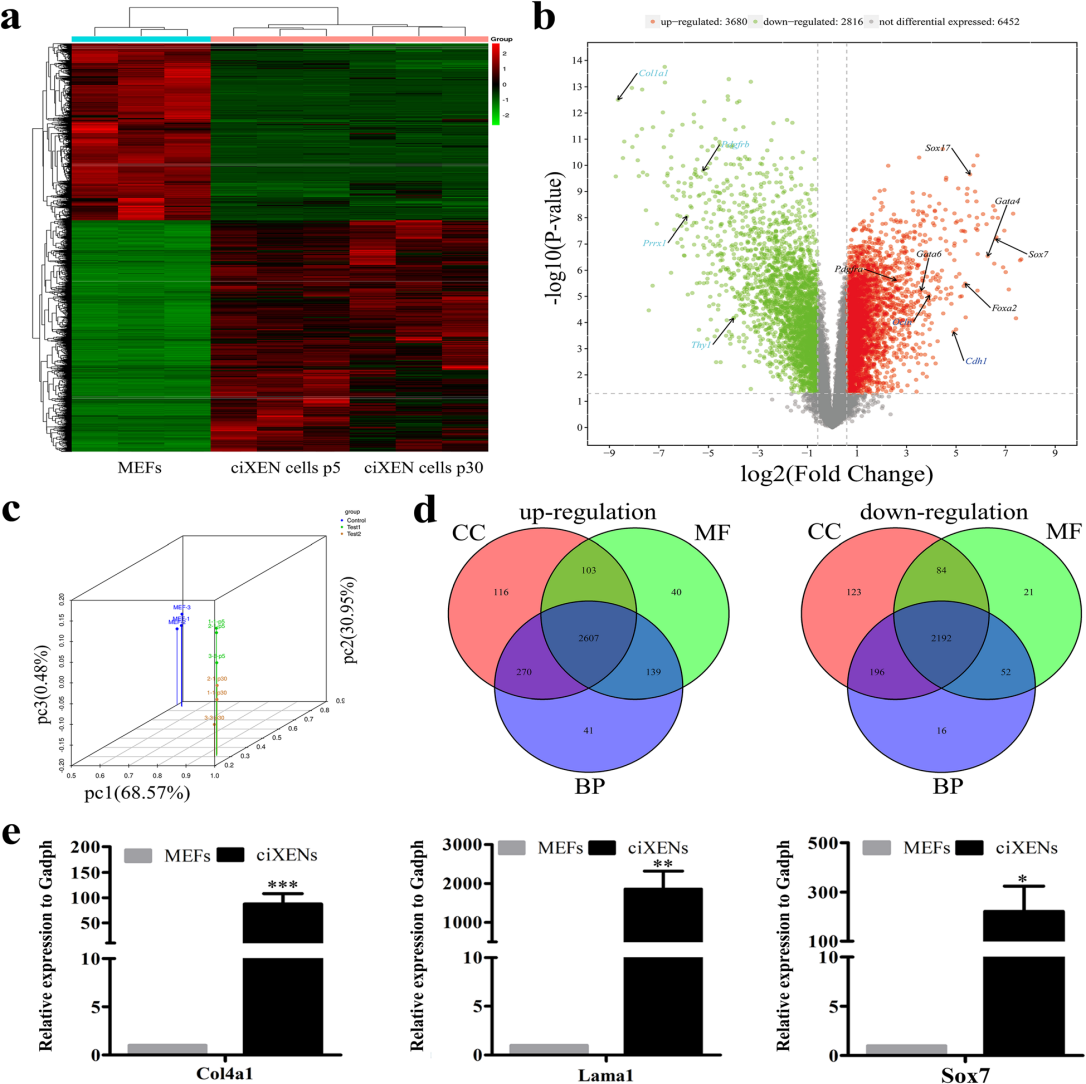
Analysis of the transcriptome of ciXEN cells. **a** Cluster analysis of DEGs in MEFs and ciXEN cells at p5 and p30. **b** Volcano plot of DEGs in MEFs and ciXEN cells at p5 and p30. The red dots represent upregulated genes, the green dots represent downregulated genes, and the grey dots represent genes without comparable expression levels. The arrows represent the XEN-related genes (*Sox17*, *Foxa2*, *Gata4*, *Gata6*, *Pdgfra*, and *Sox7*), epithelial markers (*Ocln* and *Cdh1*), and fibroblast-related genes (*Prrx1*, *Col1a1*, *Pdgfrb*, and *Thy1*). **c** PCA for the intuitive distribution of ciXEN cells and MEFs. Control, MEFs; Test 1, ciXEN cells at p5; Test 2, ciXEN cells at p30. **d** Venn diagram of GO enrichment analysis, including MF, CC, and BP. **e** Expression of other XEN markers, as determined by qPCR. *p < 0.05, **p < 0.01, ***p < 0.001. Mean ± SEM, *t* test, *n* = 3

We further analysed the transcriptome of ciXEN cells by RNA sequencing. Cluster analysis of genome-wide expression profile showed that ciXEN cells at passage 5 were analogous to those at passage 30, but the expression pattern was distinct from that observed in MEFs (Fig. 3a). Compared to MEFs, 3680 genes were upregulated, 2816 genes were downregulated, and 6452 genes exhibited no change in expression. And the volcano plot reveals that XEN markers and epithelial markers were present among the upregulated genes, while the fibroblast markers were observed among the downregulated genes (Fig. 3b). These results were consistent with the results of our previously mentioned qPCR analysis. Additionally, PCA based on genes with significant differences (*p* value ≤ 0.05) showed similarities between ciXEN cells at early and late passages (Fig. 3c). The Venn diagram of GO analysis of the upregulated and downregulated DEGs showed that three subschemas (molecular functions (MF), cellular components (CC), and biological processes (BP)) shared the same changes in gene functions (Fig. 3d). And the top ten upregulated MFs were associated with compound binding. The most dramatic changes in cellular components (CCs) were listed in Table S2 (Additional file 2) (*p* < 0.001, FDR < 0.001). Additionally, the top ten downregulated MFs were related to material binding and that downregulated CCs included those associated with the cell periphery, extracellular matrix, cell projection, and plasma membrane (Additional file 2: Table S2). We further verified the expression of other XEN markers (*Col4a1*, *Lama1*, and *Sox7*) at passage 5 by qPCR analysis (Fig. 3e). Our results indicated that the generation of ciXEN cells involved the remodelling of cell structures and functions.

### BMP4 induces ciXEN cells to differentiate into VE

**Fig. 4:**
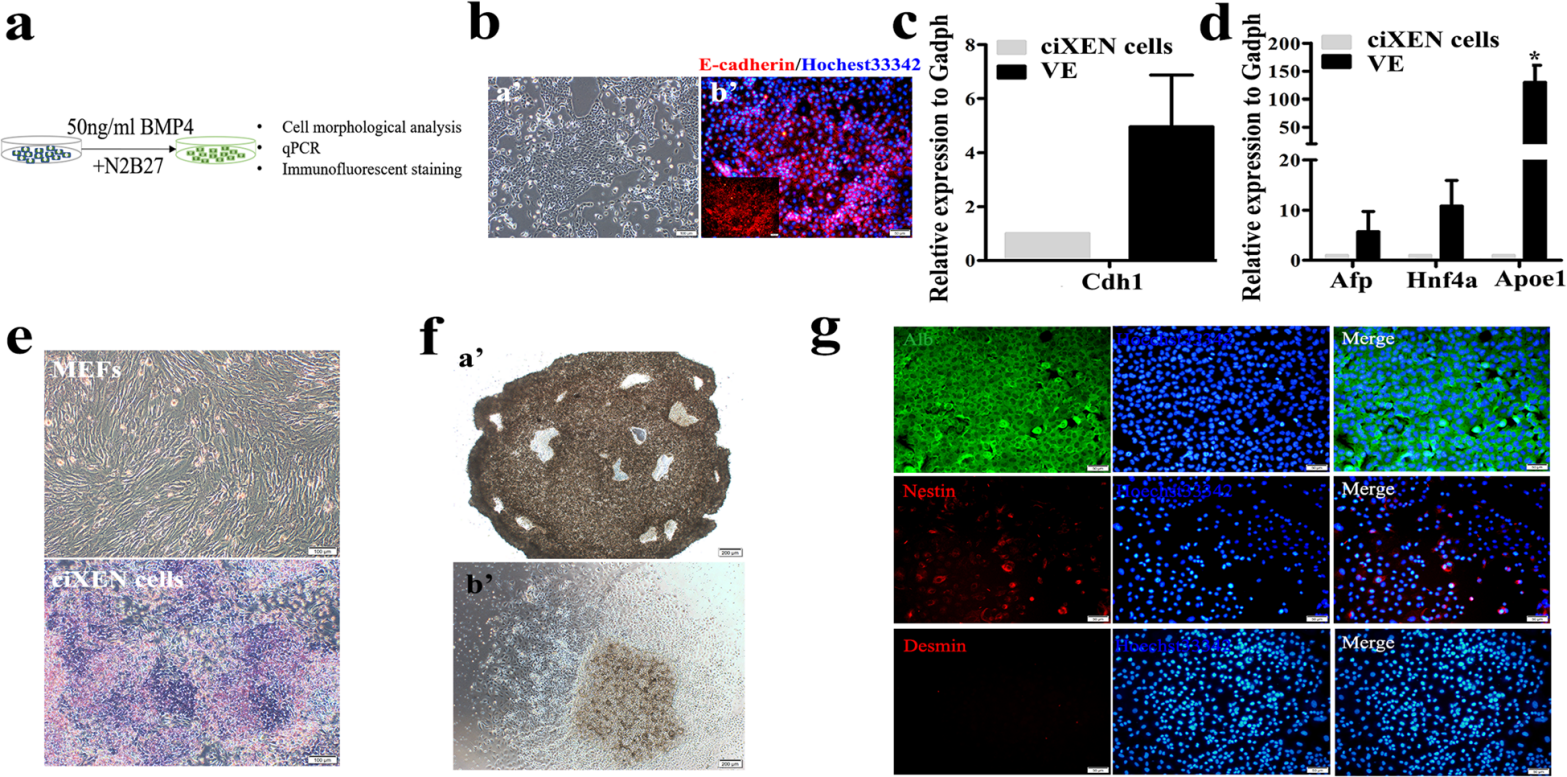
Differentiation of ciXEN cells into VE and their spontaneous differentiation. **a** The induction process of VE. **b** Cell morphologies after induction for 7 days (a’) and the immunofluorescence of E-cadherin (b’) in VE (bar, 100 μm (a’) and 50 μm (b’)). qPCR results for the expression of *Cdh1* (**c**) and VE-related genes (*Afp*, *Hnf4a*, and *Apoe1*) (**d**) in VE. **e** ALP staining of MEFs and ciXEN cells (bar, 100 μm). **f** Microscopic view of the cell mass formed after hanging-drop culture for 8 days (a’), as well as the cells that emerged from the cell mass (b’) (bar, 200 μm). **g** Immunofluorescence of Alb (endoderm), nestin (ectoderm), and desmin (mesoderm) (bar, 50 μm). * *p* < 0.05. Mean ± SEM, *t* test, *n* = 3

To induce the generation of VE, we used the optimised induction medium in accordance with the previously published report (Fig. 4a) [26]. One week later, we observed that ciXEN cells formed an epithelioid cell layer (Fig. 4b (a’)), which positively expressed E-cadherin (Fig. 4b (b’)), and they expressed *Cdh1* at a higher level than the ciXEN cells (Fig. 4c). Meanwhile, qPCR analysis showed that the expression of VE-related genes was higher than that of ciXEN cells (Fig. 4d). These results indicate that our ciXEN cells had the potency to differentiate into VE.

### Spontaneous differentiation of ciXEN cells in vitro

It has been reported that XEN progenitor cells in vitro were positive for ALP [27]. However, ALP was partially positive in our ciXEN cells (Fig. 4e), which did not express *Oct4*. This result indicates that ciXEN cells had the capacity of spontaneous differentiation that resembled pluripotent stem cells (PSCs).

Previous study has shown that XEN cells form spheres in the conditions of suspension culture [28]. However, our ciXEN cells only formed a few loose spheres. To analyse their potential to spontaneously differentiate, we cultured them using the hanging-drop method. After 8 days, the ciXEN cells formed a compact cell mass (Fig. 4f (a’)), and many cells could be observed emerging out of it (Fig. 4f (b’)). Immunofluorescence revealed that they positively expressed *Alb* (endoderm) and *Nestin* (ectoderm)—while *Desmin* (mesoderm) was not detected (Fig. 4g)—suggesting that ciXEN cells did not spontaneously differentiate into mesodermal derivatives.

### Analysing the metabolism of ciXEN cells

**Fig. 5:**
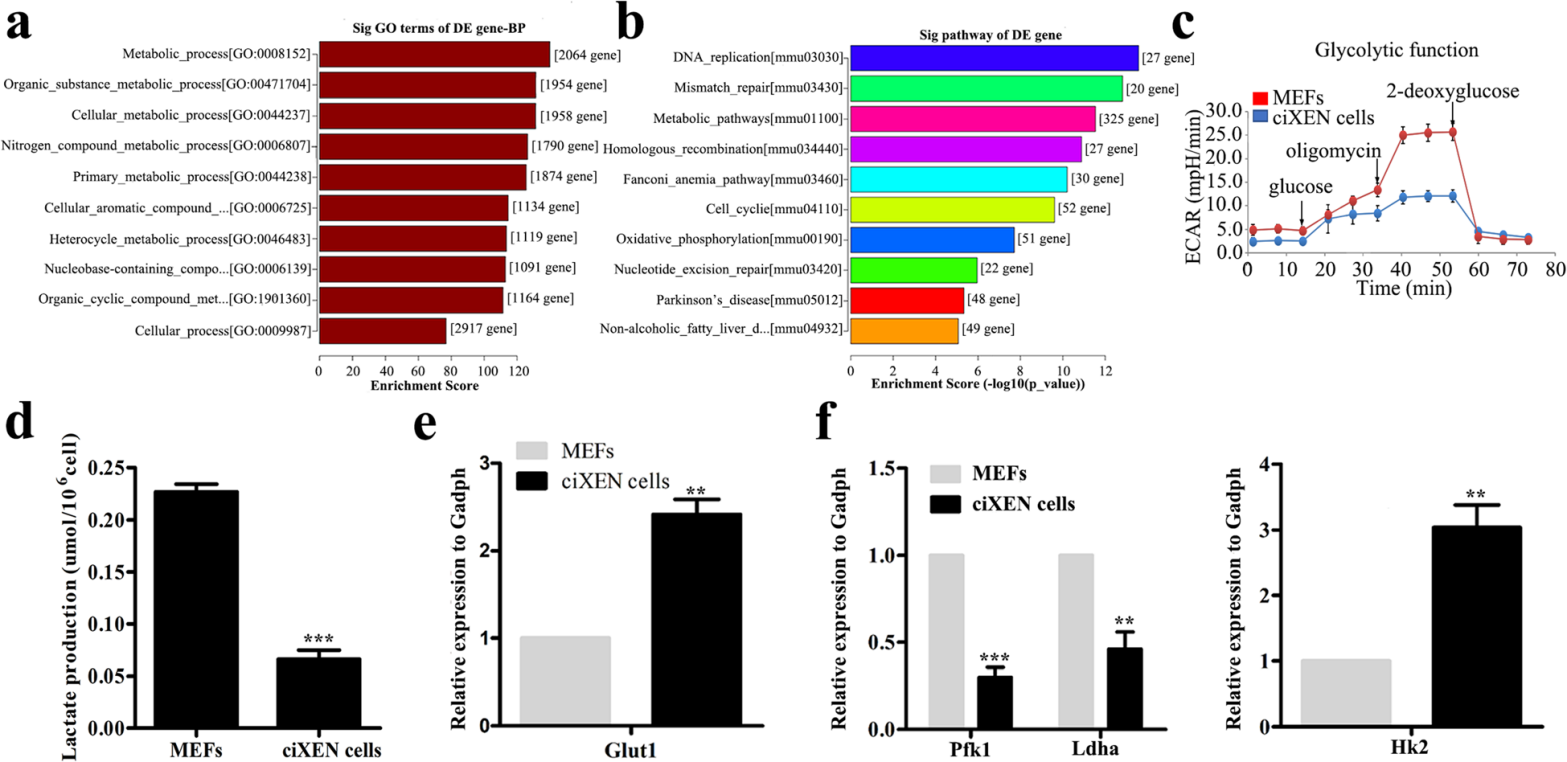
The metabolic profiles of ciXEN cells at passage 5. **a** GO analysis of the top 10 BPs based on enrichment score. **b** Pathway analysis of ciXEN cells as compared to MEFs. **c** ECAR of MEFs and ciXEN cells. **d** Lactate production of MEFs and ciXEN cells. Expression of *Glut1* (**e**) and *Pfk1*, *Ldha*, and *Hk2* (**f**) as measured by qPCR. **p < 0.01, ***p < 0.001. Mean ± SEM, *t* test, *n* = 3

In addition to transcriptome and cell functions, somatic reprogramming is accompanied by metabolic reconstruction. Nevertheless, our sequencing analysis results indicate that the top ten significantly enriched terms associated with upregulated BP of ciXEN cells were more closely related to metabolism, as compared to those of MEFs (*p* < 0.001, FDR < 0.001) (Fig. 5a). Additionally, differentially expressed metabolism-related genes were partly associated with aerobic oxidation, which was one of the top ten upregulated function parameters in KEGG analysis (Fig. 5b). But OCR analysis showed a slight decrease in maximum oxidative consumption, and total ATP level of ciXEN cells decreased (Additional file 1: Figure S3a and S3b). Other metabolic processes also were involved in upregulated pathway analysis results, including the citrate cycle and pentose phosphate pathways (Additional file 2: Table S3) (*p* < 0.05, FDR < 0.5). Compared to that in MEFs, the results of ECAR show that the glycolysis ability of ciXEN cells was not enhanced, and the production of lactate was noticeably reduced in ciXEN cells (Fig. 5c, d). These results indicated that ciXEN cells were independent of glycolysis for energy supply.

To further verify this observation, we analysed the mRNA expression of genes related to metabolism. qPCR results demonstrated that the expression of *Glut1*, a glucose transporter, was markedly upregulated in ciXEN cells, consistent with that during induction (Fig. 5e and Additional file 1: Figure S3c). The expression of *Pfk1* and *Ldha* involved in glycolysis was gradually downregulated in ciXEN cells and during chemical induction. However, the expression of *Hk2*, another gene regulating glycolysis, increased slightly (Fig. 5f and Additional file 1: Figure S3d). More importantly, the number of cell clones was not significantly affected by PS48 (Additional file 1: Figure S3e). These results further suggest that metabolic reprogramming occurred but not convert to glycolysis during the chemical-induced production of ciXEN cells.

### Differentiation of hepatocytes from ciXEN cells induced by stage cytokines

**Fig. 6:**
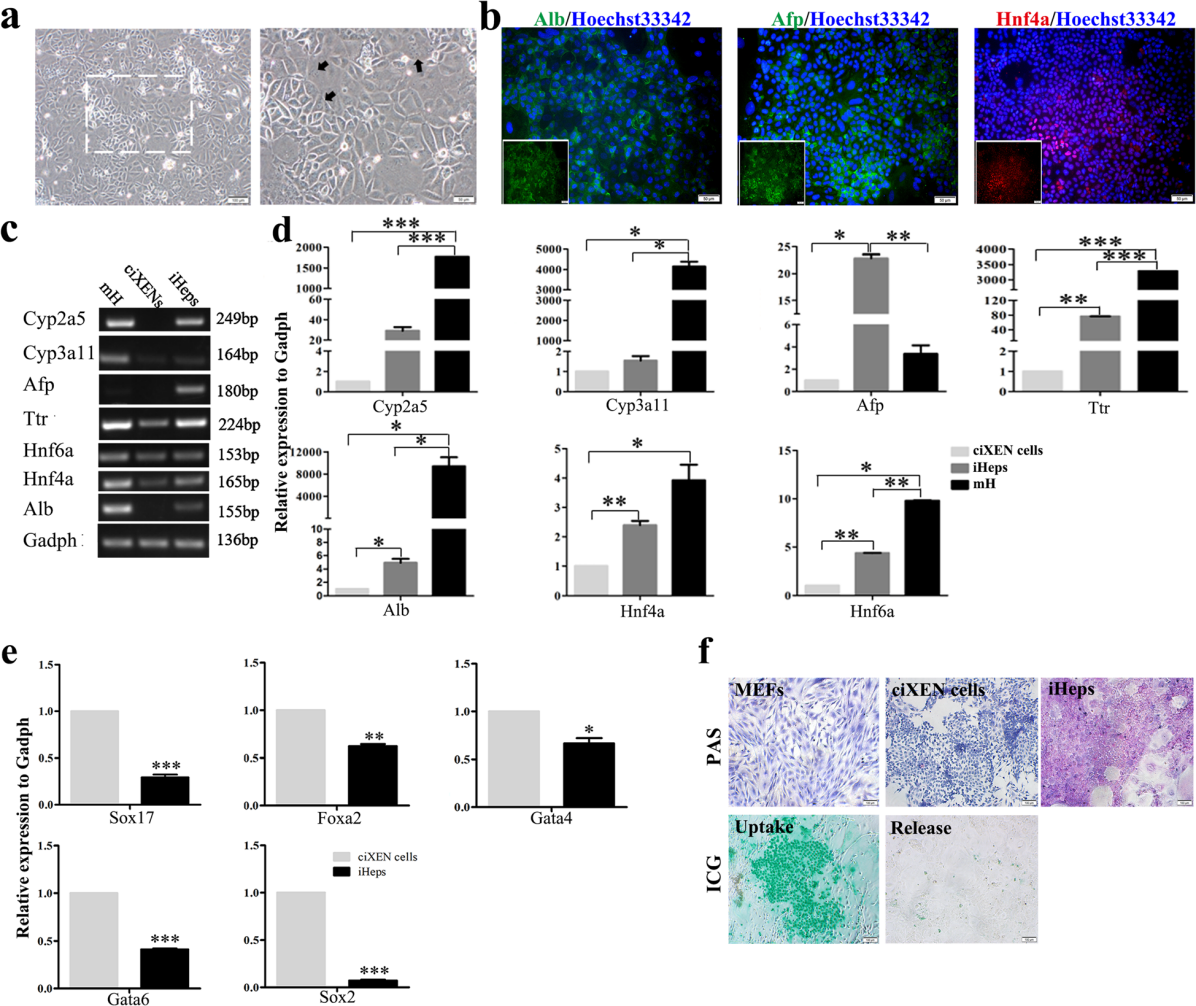
Generation of iHeps from ciXEN cells induced by staged cytokines. **a** Cell morphology of iHeps. The black arrow points to binuclear or multinuclear cells (bar, 100 μm (left) and 50 μm (right)). **b** Immunofluorescence of hepatic genes Alb, Afp, and Hnf4a in iHeps (bar, 50 μm). **c**, **d** Expression of hepatic genes detected by RT-PCR/qPCR. **e** mRNA levels of XEN genes (*Sox17*, *Gata4*, *Gata6*, and *Foxa2*) and *Sox2* in iHeps as determined by qPCR. mH, mouse hepatic tissue. **f** Functions of iHeps (glycogen storage and ICG uptake and release) detected by PAS staining and ICG assay, respectively (bar, 100 μm). *p < 0.05, **p < 0.01, ***p < 0.001. Mean ± SEM, *t* test, *n* = 3

Previous study has shown that XEN cells have high plasticity, by virtue of which they transform into definitive endodermal derivatives [12]. And our results have shown that ciXEN cells could spontaneously differentiate into endodermal cells. Therefore, we induced the differentiation of ciXEN into iHeps in accordance with our previous study [29]. After 19 days of induction with a series of staged cytokines, the cell morphologies changed to a polygonal cobblestone phenotype. In addition, some of these cells developed two or three cell nuclei (Fig. 6a). And hepatic genes, including *Afp*, *Alb*, and *Hnf4a*, were positively expressed in iHeps (Fig. 6b). PCR/qPCR analysis showed that the expression of hepatic genes increased gradually from day 0 to day 19, consistent with our qPCR analysis (Additional file 1: Figure S4). And the results of PCR/qPCR revealed highly hepatic gene expression, including *Afp*, *Alb*, *Cyp3a11*, *Cyp2a5*, *Hnf6a*, *Hnf4a*, and *Ttr* in iHeps; ciXEN cells expressed *Hnf6a*, *Hnf4a*, and *Ttr*, but their levels were lower than those in iHeps (Fig. 6c, d). Additionally, the mRNA levels of XEN-related genes and *Sox2* in iHeps were downregulated (Fig. 6e). And iHeps were capable of glycogen synthesis and storage, but ciXEN cells and MEFs did not have this ability. Additionally, ciXEN cell-derived iHeps could uptake and release ICG (Fig. 6f).

### Detecting the characteristics of iXEN cell after removing the chemicals and bFGF

Because the characteristics of ciXEN cells after removing chemicals and bFGF have not been reported, ciXEN cells were cultured in DMEM containing 1% FBS or 10% FBS (Additional file 1: Figure S5a). We discovered that cells cultured in 1% FBS medium became epithelioid, and many fibroblast-like cells were also observed (Additional file 1: Figure S5b). Additionally, their proliferative capacity was reduced due to serum restriction, which is not conducive to subsequent experiments. However, cells cultured in 10% FBS medium retained an epithelioid phenotype different from that of ciXEN cells at high density, and only a few fibroblast-like cells were present (Additional file 1: Figure S5b). After passaging, the fibroblast-like cells were not visible.

**Fig. 7:**
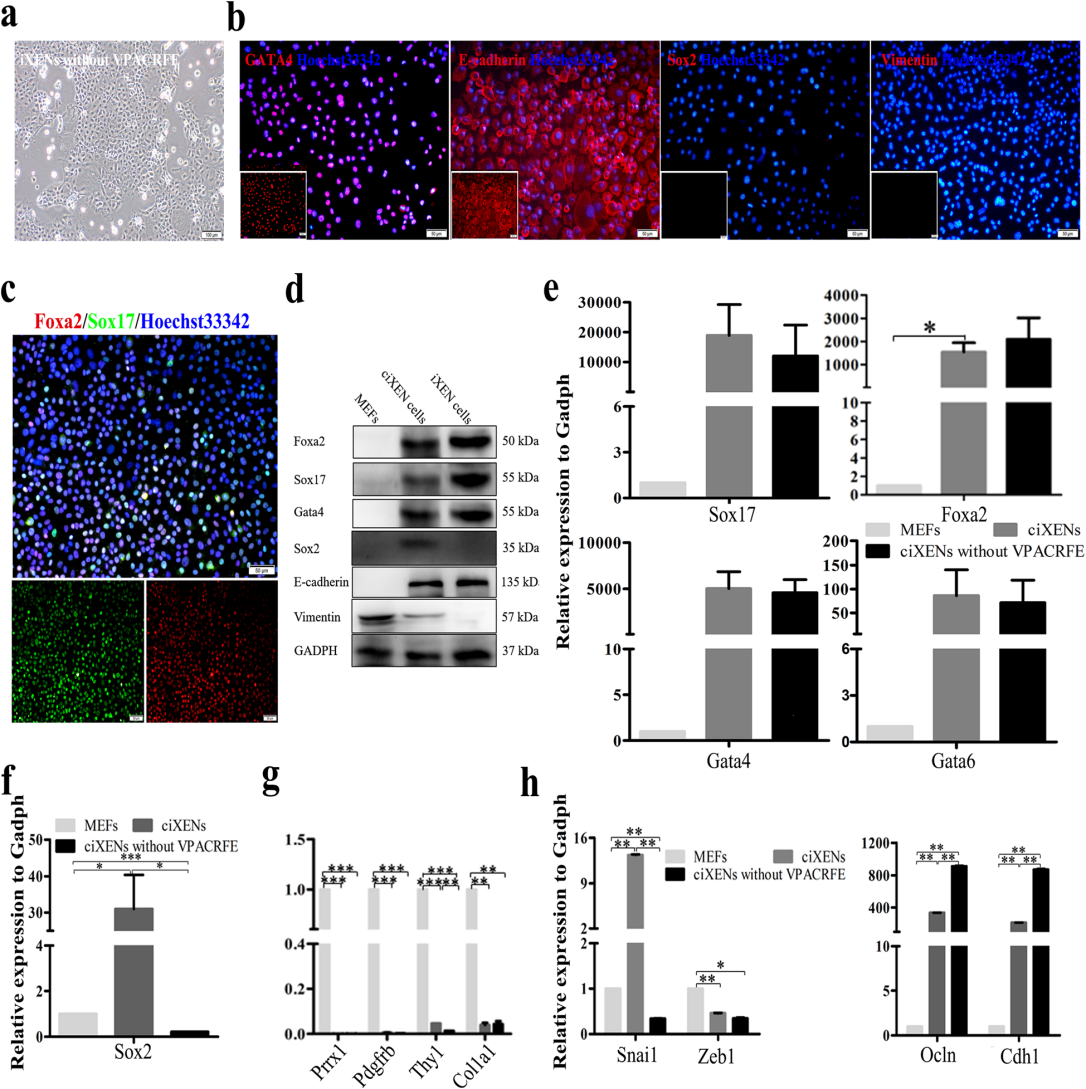
The characteristics of iXEN cells derived from ciXEN cells by withdrawing chemicals. The cell morphologies of iXEN cells (bar, 100 μm) (**a**). **b**, **c** Immunostaining of Gata4, Sox2, Foxa2, Sox17, vimentin, and E-cadherin in iXEN cells (bar, 50 μm). **d** Western blot analysis for the expression of Foxa2, Sox17, Gata4, Sox2, E-cadherin, and vimentin in MEFs, ciXEN cells, and iXEN cells. qPCR results for the expression of XEN genes (*Sox17*, *Gata4*, *Gata6*, and *Foxa2*) (**e**) and *Sox2* (**f**), fibroblast markers (*Prrx1*, *Col1a1*, *Pdgfrb*, and *Thy1*) (**g**), and epithelial genes and mesenchymal-related genes (**h**). *p < 0.05, **p < 0.01, ***p < 0.001. Mean ± SEM, two-way ANOVA, *n* = 3

Cells cultured in medium containing 10% FBS had the appearance of epithelioid after passaging (Fig. 7a), and we identified them as iXEN cells. Immunostaining indicated that they positively expressed *Gata4*, *E-cadherin*, *Sox17*, and *Foxa2*. Interestingly, they negatively expressed *Sox2* and *Vimentin* (Fig. 7b, c). These results were consistent with western blot analysis (Fig. 7d). Additionally, qPCR analysis showed that with the exception of an increase in the expression level of *Foxa2*, there was a decrease in the mRNA levels of XEN markers in iXEN cells compared to those in ciXEN cells; however, their expression levels (including *Foxa2*) were higher than those observed in MEFs (Fig. 7e). The expression of *Sox2* and the fibroblast-related genes in iXEN cells was remarkably downregulated compared to that in ciXEN cells and MEFs (Fig. 7f, g). The mRNA levels of *Cdh1* and *Ocln* were higher than those observed in the ciXEN cells and MEFs, while the levels of *Zeb1* and *Snai1* were significantly reduced (Fig. 7h). Furthermore, our EdU assay showed that the proliferative capacity of iXEN cells was significantly higher than that of MEFs, but lower than that of ciXEN cells (Additional file 1: Figure S5d).

### Generation of functional hepatocytes from iXEN cells cultured without chemicals

Additionally, we demonstrated that the cells cultured without chemicals still expressed hepatic genes (*Ttr*, *Alb*, *Hnf4a*, *Afp*, and *Cyp3a11*). However, in addition to these hepatic genes, the cells cultured in 10% FBS medium expressed *Hnf6a* (Additional file 1: Figure S5c). These results demonstrate that iXEN cells also had the capacity to differentiate into hepatocytes.

Hence, we induced iXEN cells to differentiate into hepatocytes and observed that their morphologies changed to cobblestone appearances (Additional file 1: Figure S5e). The expression levels of hepatic genes increased, while the levels of XEN genes decreased (Additional file 1: Figure S5f). And iHeps derived from iXEN cells co-expressed *Afp* and *Hnf4A*, *Asgpr1* and *E-cadherin*, and *Alb* and *Foxa3* (Additional file 1: Figure S5g). In agreement with the systemic expression of hepatic genes, iXEN cell-derived iHeps had the ability to store glycogen, as detected by PAS staining (Additional file 1: Figure S5h).

## Discussion

Researches have shown that pluripotent TFs (e.g. Sox2, Oct4, Klf4, and c-Myc) could promote the expression of PrE genes [30–33] and induce the generation of XEN-like cells from somatic cells [4, 5]. However, these protocols involve the insertion or reactivation of exogenous genes, which is associated with tumorigenic risk. Chemicals precisely manipulate cell fate conversion based on their functional reversibility and spatial and temporal controllability. Moreover, they not only improve reprogramming efficiency but also completely substitute for TFs to achieve purely chemical reprogramming, thus avoiding the risk of tumorigenesis. Chemicals commonly used in somatic reprogramming include the epigenetic modulator valproic acid (VPA, an HDAC inhibitor), signalling pathway regulators (CHIR99021, an inhibitor of GSK3-β, and RepSOX, a TGFβR-1/ALK5 inhibitor), and enzyme activity regulators (Parnate, a non-selective monoamine oxidase inhibitor; forskolin, a potent adenylate cyclase activator; EPZ004777, a selective DOT1L inhibitor; and AM580, a selective RARα agonist). At present, in addition to ciPSCs [6–9], neural stem cells [34], induced multipotent mesenchymal stem cell-like cells [35], and endoderm-like cells [36] have been successfully obtained using chemicals alone. Additionally, cells originating from different germ layers subjected to chemical reprogramming undergo the same ciXEN intermediate state [7, 10, 37]. Li et al. have shown that the chemical cocktail VPA, CHIR99021, 616452 (RepSOX), and tranylcypromine (Parnate) as a replacement for SKM (Sox2, Klf4, and Myc) enabled the reprogramming of somatic cells into iPSCs with Oct4 alone [38], which could be replaced by the synergistic effect of forskolin and DZNep in Hou’s experiments [6]. Despite its potential to substitute for Oct4, DZNep has no effect on the early stages of chemical reprogramming [6] and is therefore not applicable to our studies. As a mesenchymal lineage, the reprogramming of MEFs first requires the elimination of mesenchymal inherent signals through MET and then the reconstruction of the gene expression networks of XEN cells. During this process, VPA enhances histone acetylation, reduces epigenetic barriers to reprogramming, and improves reprogramming efficiency [39]. RepSOX, acting on TGFβR-1/ALK5, promotes the initiation of MET by inhibiting TGFβ pathways [40], and CHIR999021 mimics activation of the Wnt pathway and enhances MET in conjunction with inhibition of the TGFβ pathway, thereby accelerating the progress of hepatic reprogramming. However, activation of the Wnt pathway alone has no effect on MET [41]. As a chemical substitution of Oct4, forskolin activates cAMP-dependent pathway to facilitate gene expression via a CREB (cyclic AMP-response element-binding protein)-dependent mechanism and overcomes the transdermal barrier in reprogramming by promoting MET [42, 43]. Moreover, EPZ004777 and AM580 have been confirmed to improve reprogramming efficiency [10, 44, 45]. In addition to chemicals, FGF signalling plays an essential role during the formation of PrE by positively regulating *Gata6* while inhibiting *Nanog* [46]. Wang et al. have shown that FGFR2 expression is upregulated during the conversion of human induced endoderm progenitor cell conversion [47], and the effects of FGF9, the FGFR2 ligand, on the organic development crosstalk with canonical Wnt/β-catenin signalling [48], the activation of which facilitates reprogramming. Furthermore, bFGF promotes cell proliferation by activating the MAPK/ERK pathway [49], and hyper-proliferative cells can undergo non-random reprogramming [50]. Our results found that MEFs gradually lost their original characteristics and acquired the properties of XEN cells with the treatment of chemicals and bFGF. But more interestingly, ciXEN cells cultured in vitro without any chemicals and additional cytokines still highly expressed XEN-related genes and maintained an epithelioid phenotype, which was different from that of ciXEN cells at high density. And iXEN cells were more likely to differentiate into hepatocytes than ciXEN cells. Paracrine FGF signalling could rescue a deficiency of endogenous FGF4, which plays an important role in the generation of XEN cells from mouse ESCs, but not for the maintenance of XEN cell [51]. Our results demonstrated that the chemicals and bFGF, which are indispensable for inducing the generation of ciXEN cells, were non-essential for the in vitro culture of iXEN cells.

Except for the concentration and duration of chemicals and bFGF, the determinants of efficient reprogramming include the density of initial cells and extracellular matrix (ECM). The optimal clone efficiency was obtained from MEFs with an initial density of 4.21 × 10^3^/cm^2^, while it took 3.16 × 10^3^/cm^2^ as the optimal cell density for clone formation from MNFs. Additionally, the number of clones derived from the cells grown on the Matrigel pre-coated plates was significantly higher than that on the plates pre-coated without Matrigel. Matrigel dramatically improved the reprogramming efficiency. *Dppa5* expression significantly increases in the cells cultured with Matrigel, thus increasing the reprogramming efficiency and maintaining the pluripotency through regulation of *Nanog* [52]. And Matrigel contains many components, especially laminin, which is a major component of the basement membrane that separates the epiblast from PrE [53]. ciXEN cells in our study have two morphological appearances: refractile and epithelioid, consistent with XEN cells [54, 55], and highly expressed *Snai1*, which is not only a mesenchymal gene but a marker of PE [23]. Additionally, laminin together with BMP4 promotes MET and the formation of VE [53]. And the synergistic action of BMP4 and Matrigel induced our ciXEN cells to differentiate into VE in vitro.

Additionally, ciXEN cells cultured in vitro with chemicals and bFGF highly expressed *Sox2*, but not detected in XEN cells derived from blastocysts and PSCs. *Sox2*, which is a crucial transcription factor in PSCs and adult stem cells, is indispensable for PrE maturation. *Gata6* expression is known to decrease, while *Sox17* expression is found to be delayed in *Sox2* mutant embryos [31, 46]. In addition to *Sox2*, the expression level of gene *Sox17* in ciXEN cells was higher than that in MEFs. *Sox17* has been confirmed to be a key node in the gene regulatory networks (GRNs) of XEN cells [56], and *Sox17*-mediated XEN conversion from mouse embryonic stem cells (mESCs) is highly efficient [57]. Additionally, Sox17 overexpression in mESCs has been reported to activate both XEN and DE genetic networks [58]. However, *Sox17* is firstly upregulated when chemicals induce the generation of MEF-derived DE-like cells, which negatively express *Sox2* [36]. And after removing chemicals and bFGF, iXEN cells expressed *Sox17*, but not *Sox2*. There was no regulatory relationship between *Sox2* and *Sox17* in iXEN cells. However, how these two genes induce the production of ciXEN cell should be further explored. In addition, the presence of *Sox2* was associated with proliferative capacity [59]. The proliferative capacity of iXEN cells, which negatively expressed *Sox2*, was reduced as compared to that of ciXEN cells.

In previous studies, some overlap in the XEN and endoderm cell GRNs, such as *Sox17*, *Foxa2*, *Hnf6a*, and *Hnf4a*. With the further research on extraembryonic cells, it is found that XEN cells integrate with embryonic endoderm cells rather than replacing them [12]. Porcine PSC-derived XEN cells form embryoid bodies (EBs) with irregular margins in suspension culture, and the EBs only contain endoderm and ectoderm [28]. Additionally, PSC-derived XEN progenitor cells are positive for ALP [4]. ciXEN cells, as the first cornerstone of chemical reprogramming, bridge somatic cells to ciPSCs. And they share similar transcriptome, reprogramming potential, and developmental potential in vivo with XEN cells of blastocyst [7, 10, 11]. Despite many similarities with XEN cells, our ciXEN cells are still unique. They spontaneously differentiated into endoderm and ectoderm in a hanging-drop culture. Meanwhile, ALP could be detected in some of them. Furthermore, they expressed endodermal gene *Cxcr4*, which is negative in XEN cells. And ciXEN cells and iXEN cells both could be induced to differentiate into iHeps.

Numerous studies have verified that MET initiates reprogramming and affects reprogramming efficiency [60–63]. During the initial stages of reprogramming, promoting MET accelerates the reprogramming progress, especially for iPSCs [64, 65] and hepatocytes [41]. Our ciXEN cells maintained the expression of *Vimentin* and *Snai1*. The reason for this phenomenon involves that ciXEN cells retain the properties of the initiating cells. Incompletely reprogrammed iPSCs retain a partial memory of the initial cells during reprogramming and tend to revert to somatic cells [66–68]. This is consistent with the emergence of fibroblast-like cells after removing chemicals and bFGF. And this phenomenon is more obvious under 1% FBS condition. Additionally, iXEN cells negatively expressed *Vimentin* and *Snai1*, corresponding to our spontaneous differentiation that they were unable to differentiate into mesoderm.

In addition to gene expression and cell functions, the metabolic processes change in conjunction with the reprogramming process, but these changes, which regulate reprogramming in collaboration with epigenetics, are complex. And different cell types have different reprogramming efficiencies that may be related to the metabolic phenotype of the starting cells [69]. Our RNA sequencing results show that the significantly upregulated BP focused on metabolism. And RNA sequencing and extracellular metabolic flux analysis were consistent with those of proteomic analysis of PSC-derived XEN cells, which showed that the levels of enzymes involved in the tricarboxylic acid cycle and electron transport chain increased, while those required for mitochondrial biogenesis were downregulation [70]. Meanwhile, oxygen consumption and ATP production both decreased in ciXEN cells, which was different from the recent studies that the metabolic shift from aerobic respiration to aerobic glycolysis [71]. ciXEN cells did not depend on glycolysis for energy production, and promoting glycolysis during chemical reprogramming did not increase reprogramming efficiency, providing important insights for the study of metabolic mechanism of chemical reprogramming to improve the efficiency of reprogramming.

## Conclusions

In this study, our results revealed that ciXEN cells had high plasticity, demonstrated that their metabolic profile did not convert to glycolysis, and confirmed that chemicals and bFGF were non-essential for the in vitro culture of ciXEN cells. These results provided a powerful theoretical basis for investigating the metabolic mechanism of chemical reprogramming and established a strategy for reducing the cost of obtaining iXEN cells on a large scale.

## Supplementary information

**Additional file 1 : Figure S1.** Small molecules induced mouse fibroblasts to transform into ciXEN cells. Expression of *Epcam* (**a**) and *Cxcr4* (**b**) and mesenchymal markers (**c**) during chemical induction as measured by qPCR. **d** Western blot analysis for the expression of E-cadherin and Vimentin during chemical induction. **e** Morphological changes of MNFs induced by chemicals and bFGF (bar, 100 μm). **f** Numbers of cell clones from different numbers of initial cells: 3w, 4w, and 5w. **g** Co-immunostaining for the expression of Sox17 and Foxa2 in MNF-derived clone (bar, 50 μm). * *p* < 0.05, ** *p* < 0.01, *** *p* < 0.001. Mean ± SEM, *t*-test and two-way ANOVA analysis, *n* ≥ 3. **Figure S2.** Characteristics of ciXEN cells at different passages. qPCR results for the expression of *Epcam*, *Pdgfra*, (**a**) and *Cxcr4* (**b**) in ciXEN cells at p5. **c** The ultrastructure of MEFs and ciXEN cells. The thin arrow shows the endoplasmic reticulum (white) in MEFs and the cilium (black) in ciXEN cells; the thick arrow shows the mitochondria in ciXEN cells. (bar, 2 μm and 1 μm). **d** Cell morphologies of ciXEN cells at p5, p10, p20, p30 (bar, 100 μm). **e** Karyotype analysis of MEFs and ciXEN cells at p5, p15, and p30. mRNA levels of fibroblast genes (*Prrx1*, *Pdgfrb*, *Col1a1,* and *Thy1*), epithelial genes (*Ocln* and *Cdh1*) (**f**), XEN markers (*Sox17*, *Foxa2*, *Gata4*, and *Gata6*) (**g**), and pluripotent genes (*Oct4*, *Sox2*, and *Nanog*) (**h**). p15: passage 15; p20: passage 20. * *p* < 0.05, ** *p* < 0.01, *** *p* < 0.001. Mean ± SEM, *t*-test and two-way ANOVA analysis, *n* = 3. **Figure S3.** The metabolic patterns of ciXEN cells. **a** OCR of MEFs and ciXEN cells. **b** Total ATP of MEFs and ciXEN cells. Expression of *Glut1* (**c**) and *Pfk1*, *Ldha*, and *Hk2* (**d**) during chemical induction as measured by qPCR. **e** Numbers of cell clones under different treatment conditions: MEFs + VPACRFE and MEFs + VPACRFE + PS48. * *p* < 0.05, ** *p* < 0.01, *** *p* < 0.001. Mean ± SEM, *t*-test, n = 3. **Figure S4.** PCR/qPCR analysis for the expression of hepatic genes during the induction of iHeps. * *p* < 0.05, ** *p* < 0.01, *** *p* < 0.001. Mean ± SEM, *t*-test, n = 3. **Figure S5.** The characteristics of iXEN cells and their potential to differentiate into iHeps. **a** Experimental procedures of ciXEN cells cultured in different conditions: 1% FBS and 10% FBS. **b** The morphologies of the cells cultured in the medium containing 1% FBS (left) and 10% FBS (right) (bar, 50 μm). **c** qPCR results for the expression of hepatic markers in the cells with 1% FBS and 10% FBS. **d** EdU assay for the proliferative ability of MEFs, ciXEN cells and iXEN cells (bar, 50 μm). **e** Morphology of iHeps induced from iXEN cells (bar, 100 μm). **f** mRNA levels of hepatic genes, XEN genes (*Sox17*, *Foxa2*, *Gata4*, and *Gata6*) and *Sox2*, as measured by qPCR. **g** Co-immunostaining for the expression of Afp and Hnf4a, Alb and Foxa3, Asgpr1 and E-cadherin (bar, 50 μm). **h** PAS staining of iXEN cell-derived iHeps (bar, 100 μm). * *p* < 0.05, ** *p* < 0.01, *** *p* < 0.001. Mean ± SEM, *t*-test, n = 3.

**Additional file 2 : Table S1.** Primers used for PCR/qPCR. **Table S2.** GO analysis of the top 10 upregulated CCs and MFs, and the top 10 downregulated CCs and MFs in ciXEN cells at passage 5 and passage 30 compared to those in MEFs. **Table S3.** Pathway analysis of the upregulated metabolic pathways in ciXEN cells at passage 5 compared to those in MEFs.

## Data Availability

All data generated or analysed during this study are included in this published article.

## Abbreviations

ciXEN cells: Chemical-induced extraembryonic endoderm-like cells
MEFs: Mouse embryonic fibroblasts
XEN: Extraembryonic endoderm
iHeps: Induced hepatocytes
iXEN cells: Induced extraembryonic endoderm-like cells
TF: Transcription factor
iPSCs: Induced pluripotent stem cells
ciPSCs: Chemical-induced pluripotent stem cells
PrE: Primary endoderm
PE: Parietal endoderm
DE: Definitive endoderm
MNFs: Mouse neonatal fibroblasts
FCM: Fibroblast culture medium
FBS: Foetal bovine serum
IM: Induced medium
MM: Maintenance medium
VE: Visceral endoderm
NEAA: Non-essential amino acids
HIM I: Hepatic induced medium I
HIM II: Hepatic induced medium II
DEGs: Differentially expressed genes
FPKM: Kilobase of transcript per million mapped
PCA: Principal component analysis
GO: Gene Ontology
KEGG: Kyoto Encyclopaedia of Genes and Genomes
PFA: Paraformaldehyde
PVDF: Polyvinylidene fluoride
PI: Proliferation index
TEM: Transmission electron microscopy
ALP: Alkaline phosphatase
PAS: Periodic acid-Schiff
ddH_2_O: Double distilled water
ICG: Indocyanine green
ECAR: Extracellular acidification rate
OCR: Oxygen consumption rate
ATP: Adenosine triphosphate
MET: Mesenchymal epithelial transition
MF: Molecular functions
CC: Cellular components
BP: Biological processes
PSCs: Pluripotent stem cells
VPA: Valproic acid
ECM: Extracellular matrix
GRNs: Gene regulatory networks
mESCs: Mouse embryonic stem cells
EBs: Embryoid bodies
