# Supplementary figures and images for: Characterisation of extraembryonic endoderm-like cells from mouse embryonic fibroblasts induced using chemicals alone
Source: Stem Cell Res Ther. 2020 Apr 16;11:157. doi: 10.1186/s13287-020-01664-0 (PMC7164364; doi:10.1186/s13287-020-01664-0)

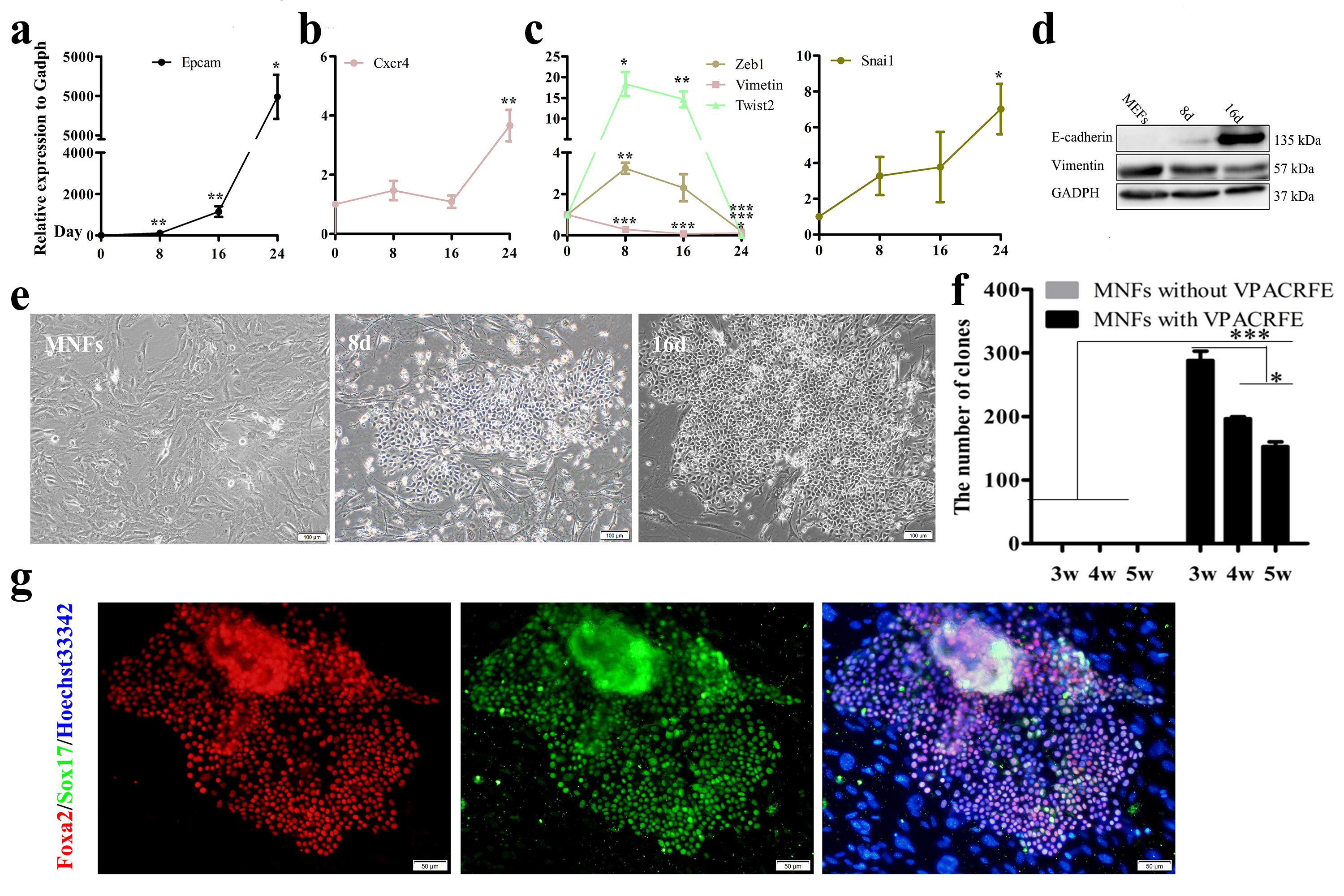

Supplement: Supplementary file 1 — Additional file 1 : Figure S1. Small molecules induced mouse fibroblasts to transform into ciXEN cells. Expression of Epcam (a) and Cxcr4 (b) and mesenchymal markers (c) during chemical induction as measured by qPCR. d Western blot analysis for the expression of E-cadherin and Vimentin during chemical induction. e Morphological changes of MNFs induced by chemicals and bFGF (bar, 100 μm). f Numbers of cell clones from different numbers of initial cells: 3w, 4w, and 5w. g Co-immunostaining for the expression of Sox17 and Foxa2 in MNF-derived clone (bar, 50 μm). * p < 0.05, ** p < 0.01, *** p < 0.001. Mean ± SEM, t-test and two-way ANOVA analysis, n ≥ 3. Figure S2. Characteristics of ciXEN cells at different passages. qPCR results for the expression of Epcam, Pdgfra, (a) and Cxcr4 (b) in ciXEN cells at p5. c The ultrastructure of MEFs and ciXEN cells. The thin arrow shows the endoplasmic reticulum (white) in MEFs and the cilium (black) in ciXEN cells; the thick arrow shows the mitochondria in ciXEN cells. (bar, 2 μm and 1 μm). d Cell morphologies of ciXEN cells at p5, p10, p20, p30 (bar, 100 μm). e Karyotype analysis of MEFs and ciXEN cells at p5, p15, and p30. mRNA levels of fibroblast genes (Prrx1, Pdgfrb, Col1a1, and Thy1), epithelial genes (Ocln and Cdh1) (f), XEN markers (Sox17, Foxa2, Gata4, and Gata6) (g), and pluripotent genes (Oct4, Sox2, and Nanog) (h). p15: passage 15; p20: passage 20. * p < 0.05, ** p < 0.01, *** p < 0.001. Mean ± SEM, t-test and two-way ANOVA analysis, n = 3. Figure S3. The metabolic patterns of ciXEN cells. a OCR of MEFs and ciXEN cells. b Total ATP of MEFs and ciXEN cells. Expression of Glut1 (c) and Pfk1, Ldha, and Hk2 (d) during chemical induction as measured by qPCR. e Numbers of cell clones under different treatment conditions: MEFs + VPACRFE and MEFs + VPACRFE + PS48. * p < 0.05, ** p < 0.01, *** p < 0.001. Mean ± SEM, t-test, n = 3. Figure S4. PCR/qPCR analysis for the expression of hepatic genes during the induction of iHeps. * p [file 13287_2020_1664_MOESM1_ESM.zip › Figure S1.tif]

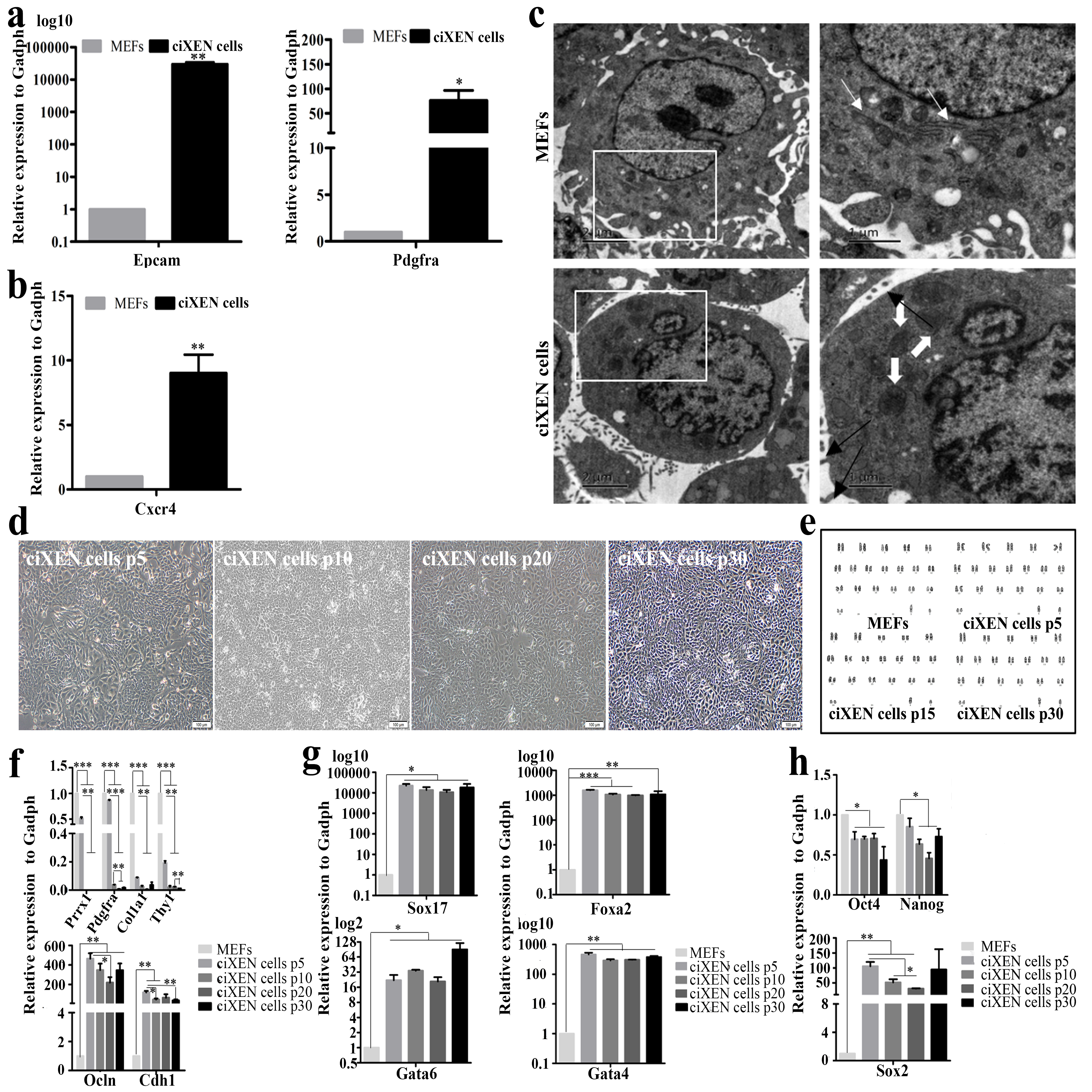

Supplement: Supplementary file 1 — Additional file 1 : Figure S1. Small molecules induced mouse fibroblasts to transform into ciXEN cells. Expression of Epcam (a) and Cxcr4 (b) and mesenchymal markers (c) during chemical induction as measured by qPCR. d Western blot analysis for the expression of E-cadherin and Vimentin during chemical induction. e Morphological changes of MNFs induced by chemicals and bFGF (bar, 100 μm). f Numbers of cell clones from different numbers of initial cells: 3w, 4w, and 5w. g Co-immunostaining for the expression of Sox17 and Foxa2 in MNF-derived clone (bar, 50 μm). * p < 0.05, ** p < 0.01, *** p < 0.001. Mean ± SEM, t-test and two-way ANOVA analysis, n ≥ 3. Figure S2. Characteristics of ciXEN cells at different passages. qPCR results for the expression of Epcam, Pdgfra, (a) and Cxcr4 (b) in ciXEN cells at p5. c The ultrastructure of MEFs and ciXEN cells. The thin arrow shows the endoplasmic reticulum (white) in MEFs and the cilium (black) in ciXEN cells; the thick arrow shows the mitochondria in ciXEN cells. (bar, 2 μm and 1 μm). d Cell morphologies of ciXEN cells at p5, p10, p20, p30 (bar, 100 μm). e Karyotype analysis of MEFs and ciXEN cells at p5, p15, and p30. mRNA levels of fibroblast genes (Prrx1, Pdgfrb, Col1a1, and Thy1), epithelial genes (Ocln and Cdh1) (f), XEN markers (Sox17, Foxa2, Gata4, and Gata6) (g), and pluripotent genes (Oct4, Sox2, and Nanog) (h). p15: passage 15; p20: passage 20. * p < 0.05, ** p < 0.01, *** p < 0.001. Mean ± SEM, t-test and two-way ANOVA analysis, n = 3. Figure S3. The metabolic patterns of ciXEN cells. a OCR of MEFs and ciXEN cells. b Total ATP of MEFs and ciXEN cells. Expression of Glut1 (c) and Pfk1, Ldha, and Hk2 (d) during chemical induction as measured by qPCR. e Numbers of cell clones under different treatment conditions: MEFs + VPACRFE and MEFs + VPACRFE + PS48. * p < 0.05, ** p < 0.01, *** p < 0.001. Mean ± SEM, t-test, n = 3. Figure S4. PCR/qPCR analysis for the expression of hepatic genes during the induction of iHeps. * p [file 13287_2020_1664_MOESM1_ESM.zip › Figure S2.tif]

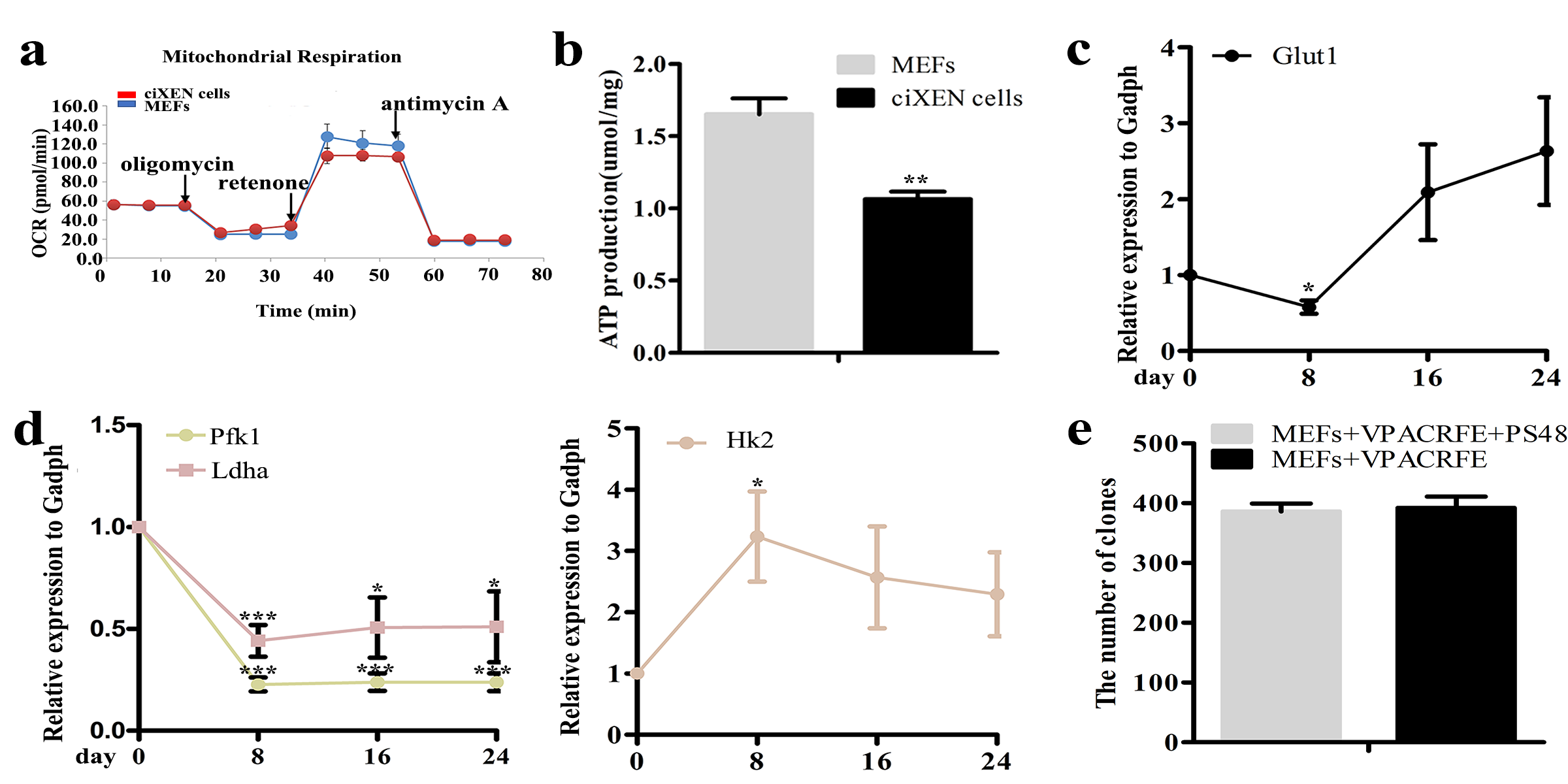

Supplement: Supplementary file 1 — Additional file 1 : Figure S1. Small molecules induced mouse fibroblasts to transform into ciXEN cells. Expression of Epcam (a) and Cxcr4 (b) and mesenchymal markers (c) during chemical induction as measured by qPCR. d Western blot analysis for the expression of E-cadherin and Vimentin during chemical induction. e Morphological changes of MNFs induced by chemicals and bFGF (bar, 100 μm). f Numbers of cell clones from different numbers of initial cells: 3w, 4w, and 5w. g Co-immunostaining for the expression of Sox17 and Foxa2 in MNF-derived clone (bar, 50 μm). * p < 0.05, ** p < 0.01, *** p < 0.001. Mean ± SEM, t-test and two-way ANOVA analysis, n ≥ 3. Figure S2. Characteristics of ciXEN cells at different passages. qPCR results for the expression of Epcam, Pdgfra, (a) and Cxcr4 (b) in ciXEN cells at p5. c The ultrastructure of MEFs and ciXEN cells. The thin arrow shows the endoplasmic reticulum (white) in MEFs and the cilium (black) in ciXEN cells; the thick arrow shows the mitochondria in ciXEN cells. (bar, 2 μm and 1 μm). d Cell morphologies of ciXEN cells at p5, p10, p20, p30 (bar, 100 μm). e Karyotype analysis of MEFs and ciXEN cells at p5, p15, and p30. mRNA levels of fibroblast genes (Prrx1, Pdgfrb, Col1a1, and Thy1), epithelial genes (Ocln and Cdh1) (f), XEN markers (Sox17, Foxa2, Gata4, and Gata6) (g), and pluripotent genes (Oct4, Sox2, and Nanog) (h). p15: passage 15; p20: passage 20. * p < 0.05, ** p < 0.01, *** p < 0.001. Mean ± SEM, t-test and two-way ANOVA analysis, n = 3. Figure S3. The metabolic patterns of ciXEN cells. a OCR of MEFs and ciXEN cells. b Total ATP of MEFs and ciXEN cells. Expression of Glut1 (c) and Pfk1, Ldha, and Hk2 (d) during chemical induction as measured by qPCR. e Numbers of cell clones under different treatment conditions: MEFs + VPACRFE and MEFs + VPACRFE + PS48. * p < 0.05, ** p < 0.01, *** p < 0.001. Mean ± SEM, t-test, n = 3. Figure S4. PCR/qPCR analysis for the expression of hepatic genes during the induction of iHeps. * p [file 13287_2020_1664_MOESM1_ESM.zip › Figure S3.tif]

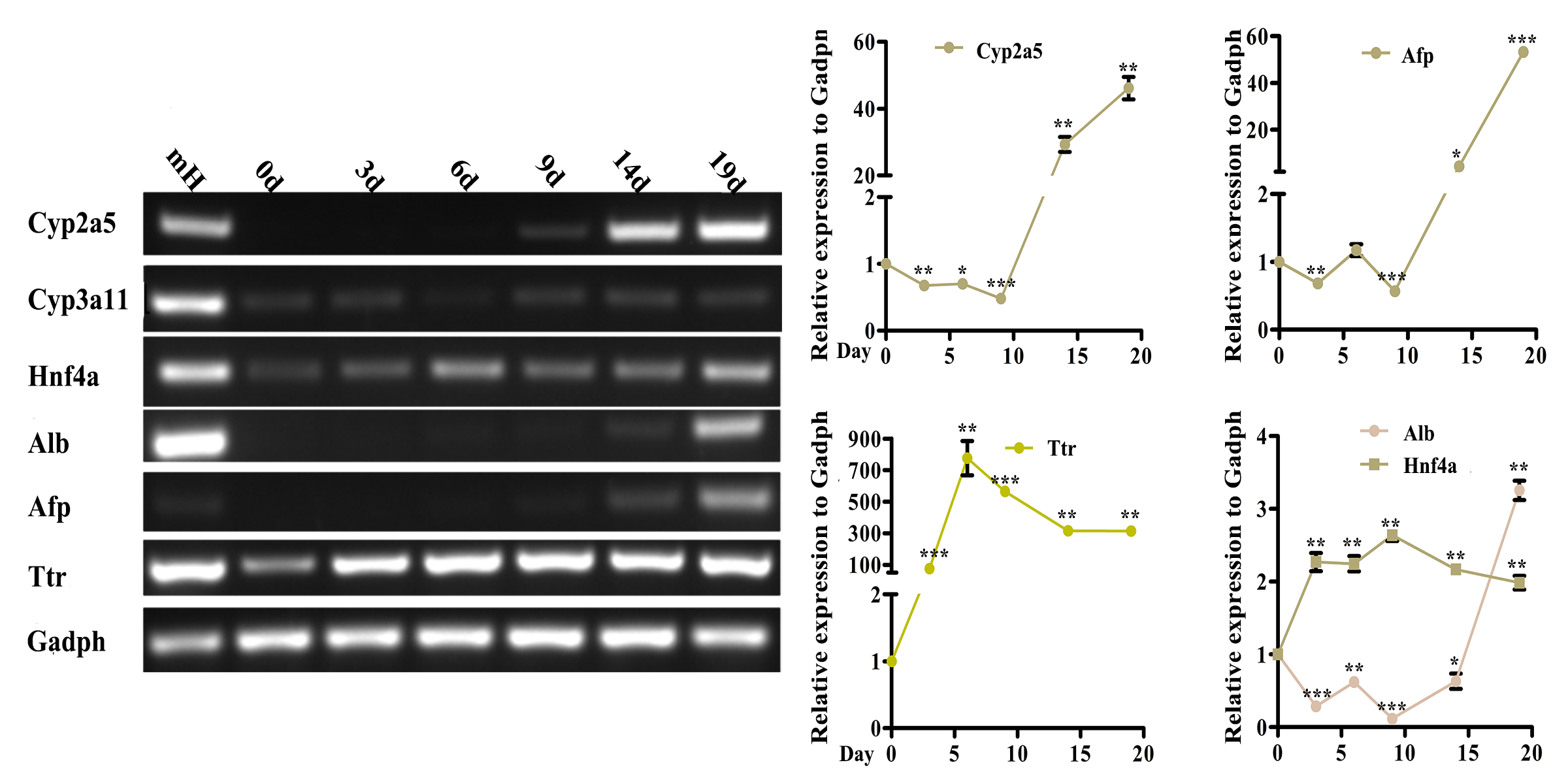

Supplement: Supplementary file 1 — Additional file 1 : Figure S1. Small molecules induced mouse fibroblasts to transform into ciXEN cells. Expression of Epcam (a) and Cxcr4 (b) and mesenchymal markers (c) during chemical induction as measured by qPCR. d Western blot analysis for the expression of E-cadherin and Vimentin during chemical induction. e Morphological changes of MNFs induced by chemicals and bFGF (bar, 100 μm). f Numbers of cell clones from different numbers of initial cells: 3w, 4w, and 5w. g Co-immunostaining for the expression of Sox17 and Foxa2 in MNF-derived clone (bar, 50 μm). * p < 0.05, ** p < 0.01, *** p < 0.001. Mean ± SEM, t-test and two-way ANOVA analysis, n ≥ 3. Figure S2. Characteristics of ciXEN cells at different passages. qPCR results for the expression of Epcam, Pdgfra, (a) and Cxcr4 (b) in ciXEN cells at p5. c The ultrastructure of MEFs and ciXEN cells. The thin arrow shows the endoplasmic reticulum (white) in MEFs and the cilium (black) in ciXEN cells; the thick arrow shows the mitochondria in ciXEN cells. (bar, 2 μm and 1 μm). d Cell morphologies of ciXEN cells at p5, p10, p20, p30 (bar, 100 μm). e Karyotype analysis of MEFs and ciXEN cells at p5, p15, and p30. mRNA levels of fibroblast genes (Prrx1, Pdgfrb, Col1a1, and Thy1), epithelial genes (Ocln and Cdh1) (f), XEN markers (Sox17, Foxa2, Gata4, and Gata6) (g), and pluripotent genes (Oct4, Sox2, and Nanog) (h). p15: passage 15; p20: passage 20. * p < 0.05, ** p < 0.01, *** p < 0.001. Mean ± SEM, t-test and two-way ANOVA analysis, n = 3. Figure S3. The metabolic patterns of ciXEN cells. a OCR of MEFs and ciXEN cells. b Total ATP of MEFs and ciXEN cells. Expression of Glut1 (c) and Pfk1, Ldha, and Hk2 (d) during chemical induction as measured by qPCR. e Numbers of cell clones under different treatment conditions: MEFs + VPACRFE and MEFs + VPACRFE + PS48. * p < 0.05, ** p < 0.01, *** p < 0.001. Mean ± SEM, t-test, n = 3. Figure S4. PCR/qPCR analysis for the expression of hepatic genes during the induction of iHeps. * p [file 13287_2020_1664_MOESM1_ESM.zip › Figure S4.tif]

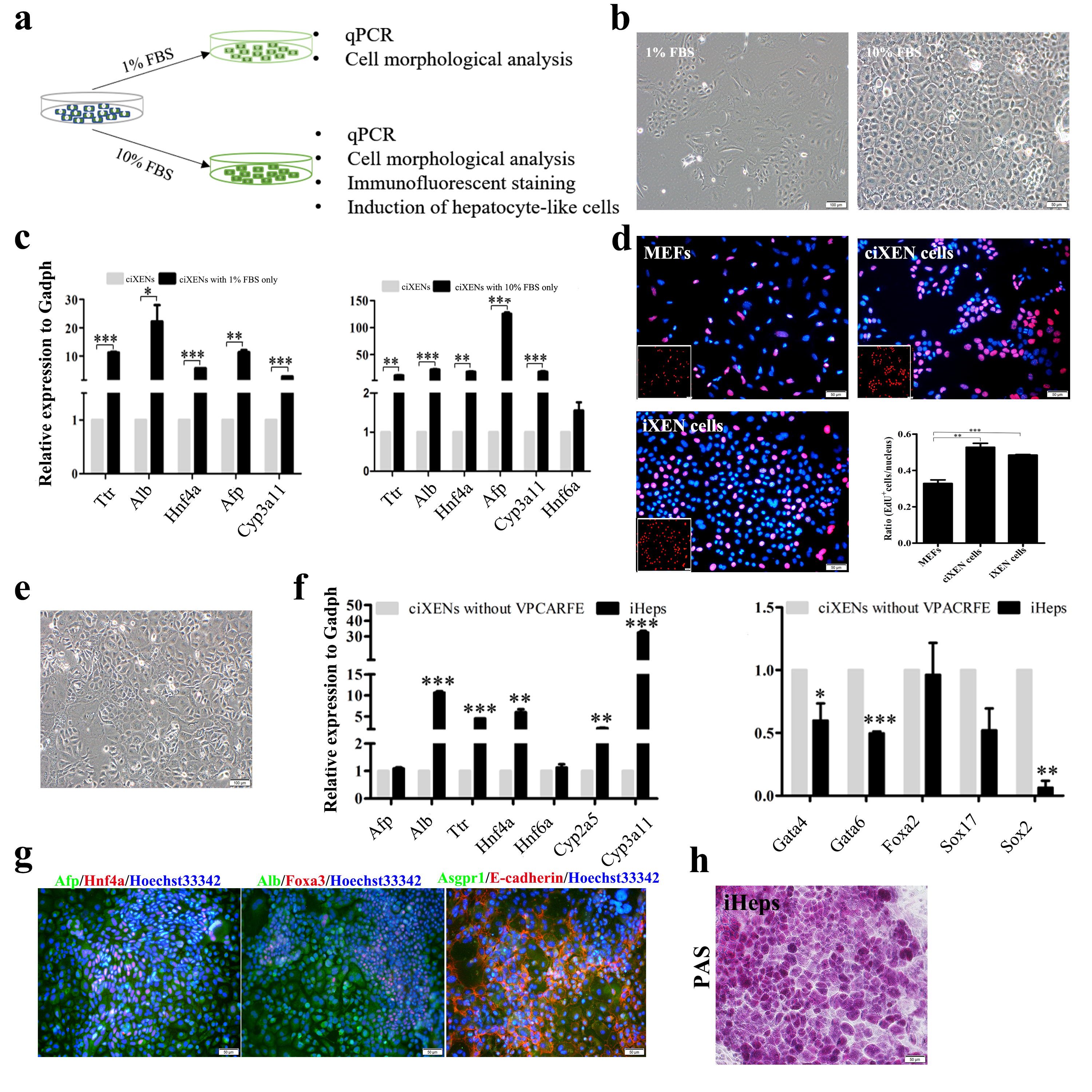

Supplement: Supplementary file 1 — Additional file 1 : Figure S1. Small molecules induced mouse fibroblasts to transform into ciXEN cells. Expression of Epcam (a) and Cxcr4 (b) and mesenchymal markers (c) during chemical induction as measured by qPCR. d Western blot analysis for the expression of E-cadherin and Vimentin during chemical induction. e Morphological changes of MNFs induced by chemicals and bFGF (bar, 100 μm). f Numbers of cell clones from different numbers of initial cells: 3w, 4w, and 5w. g Co-immunostaining for the expression of Sox17 and Foxa2 in MNF-derived clone (bar, 50 μm). * p < 0.05, ** p < 0.01, *** p < 0.001. Mean ± SEM, t-test and two-way ANOVA analysis, n ≥ 3. Figure S2. Characteristics of ciXEN cells at different passages. qPCR results for the expression of Epcam, Pdgfra, (a) and Cxcr4 (b) in ciXEN cells at p5. c The ultrastructure of MEFs and ciXEN cells. The thin arrow shows the endoplasmic reticulum (white) in MEFs and the cilium (black) in ciXEN cells; the thick arrow shows the mitochondria in ciXEN cells. (bar, 2 μm and 1 μm). d Cell morphologies of ciXEN cells at p5, p10, p20, p30 (bar, 100 μm). e Karyotype analysis of MEFs and ciXEN cells at p5, p15, and p30. mRNA levels of fibroblast genes (Prrx1, Pdgfrb, Col1a1, and Thy1), epithelial genes (Ocln and Cdh1) (f), XEN markers (Sox17, Foxa2, Gata4, and Gata6) (g), and pluripotent genes (Oct4, Sox2, and Nanog) (h). p15: passage 15; p20: passage 20. * p < 0.05, ** p < 0.01, *** p < 0.001. Mean ± SEM, t-test and two-way ANOVA analysis, n = 3. Figure S3. The metabolic patterns of ciXEN cells. a OCR of MEFs and ciXEN cells. b Total ATP of MEFs and ciXEN cells. Expression of Glut1 (c) and Pfk1, Ldha, and Hk2 (d) during chemical induction as measured by qPCR. e Numbers of cell clones under different treatment conditions: MEFs + VPACRFE and MEFs + VPACRFE + PS48. * p < 0.05, ** p < 0.01, *** p < 0.001. Mean ± SEM, t-test, n = 3. Figure S4. PCR/qPCR analysis for the expression of hepatic genes during the induction of iHeps. * p [file 13287_2020_1664_MOESM1_ESM.zip › figure S5.tif]

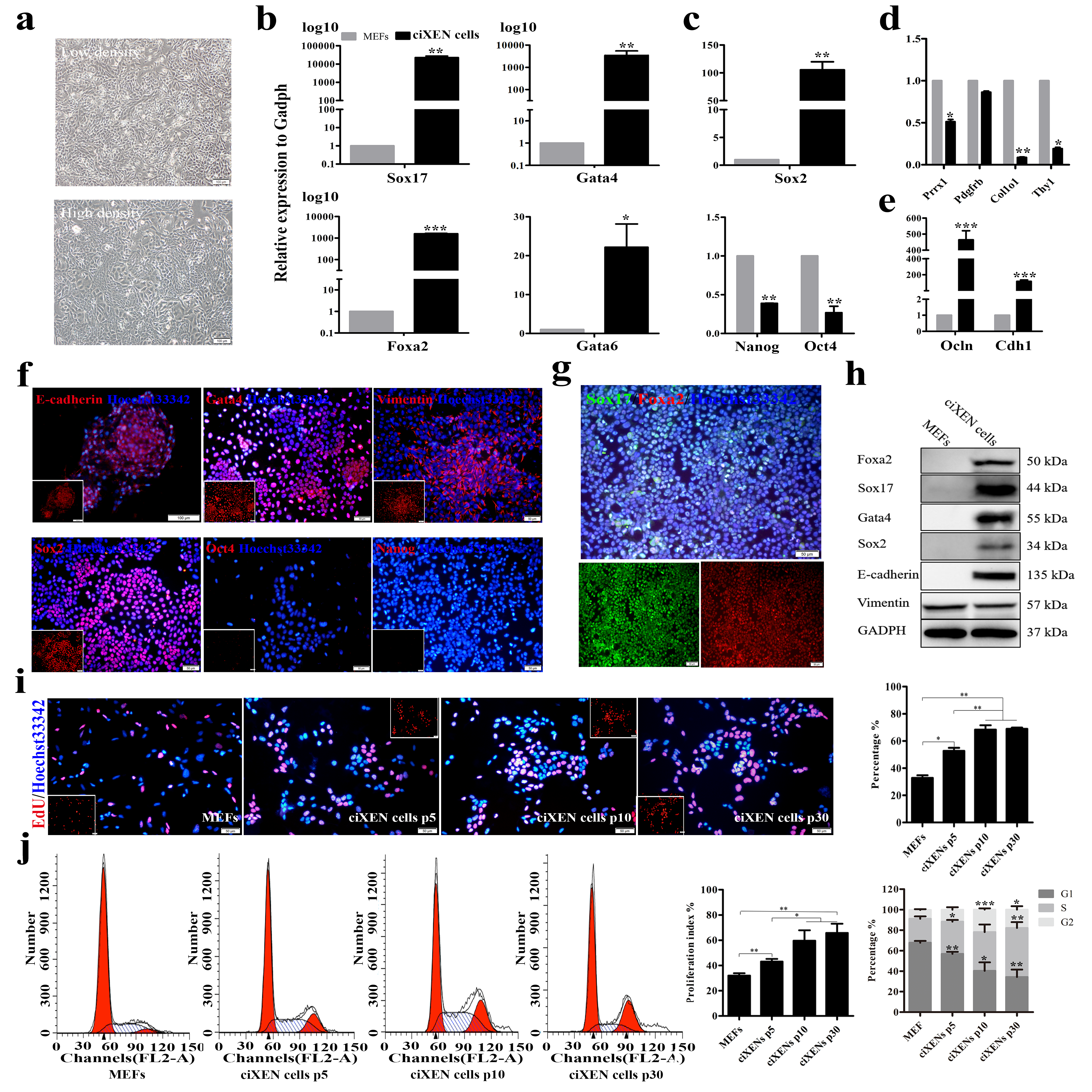

Supplement: Supplementary file 1 — Additional file 1 : Figure S1. Small molecules induced mouse fibroblasts to transform into ciXEN cells. Expression of Epcam (a) and Cxcr4 (b) and mesenchymal markers (c) during chemical induction as measured by qPCR. d Western blot analysis for the expression of E-cadherin and Vimentin during chemical induction. e Morphological changes of MNFs induced by chemicals and bFGF (bar, 100 μm). f Numbers of cell clones from different numbers of initial cells: 3w, 4w, and 5w. g Co-immunostaining for the expression of Sox17 and Foxa2 in MNF-derived clone (bar, 50 μm). * p < 0.05, ** p < 0.01, *** p < 0.001. Mean ± SEM, t-test and two-way ANOVA analysis, n ≥ 3. Figure S2. Characteristics of ciXEN cells at different passages. qPCR results for the expression of Epcam, Pdgfra, (a) and Cxcr4 (b) in ciXEN cells at p5. c The ultrastructure of MEFs and ciXEN cells. The thin arrow shows the endoplasmic reticulum (white) in MEFs and the cilium (black) in ciXEN cells; the thick arrow shows the mitochondria in ciXEN cells. (bar, 2 μm and 1 μm). d Cell morphologies of ciXEN cells at p5, p10, p20, p30 (bar, 100 μm). e Karyotype analysis of MEFs and ciXEN cells at p5, p15, and p30. mRNA levels of fibroblast genes (Prrx1, Pdgfrb, Col1a1, and Thy1), epithelial genes (Ocln and Cdh1) (f), XEN markers (Sox17, Foxa2, Gata4, and Gata6) (g), and pluripotent genes (Oct4, Sox2, and Nanog) (h). p15: passage 15; p20: passage 20. * p < 0.05, ** p < 0.01, *** p < 0.001. Mean ± SEM, t-test and two-way ANOVA analysis, n = 3. Figure S3. The metabolic patterns of ciXEN cells. a OCR of MEFs and ciXEN cells. b Total ATP of MEFs and ciXEN cells. Expression of Glut1 (c) and Pfk1, Ldha, and Hk2 (d) during chemical induction as measured by qPCR. e Numbers of cell clones under different treatment conditions: MEFs + VPACRFE and MEFs + VPACRFE + PS48. * p < 0.05, ** p < 0.01, *** p < 0.001. Mean ± SEM, t-test, n = 3. Figure S4. PCR/qPCR analysis for the expression of hepatic genes during the induction of iHeps. * p [file 13287_2020_1664_MOESM1_ESM.zip › updated figures/Fig.2.tif]

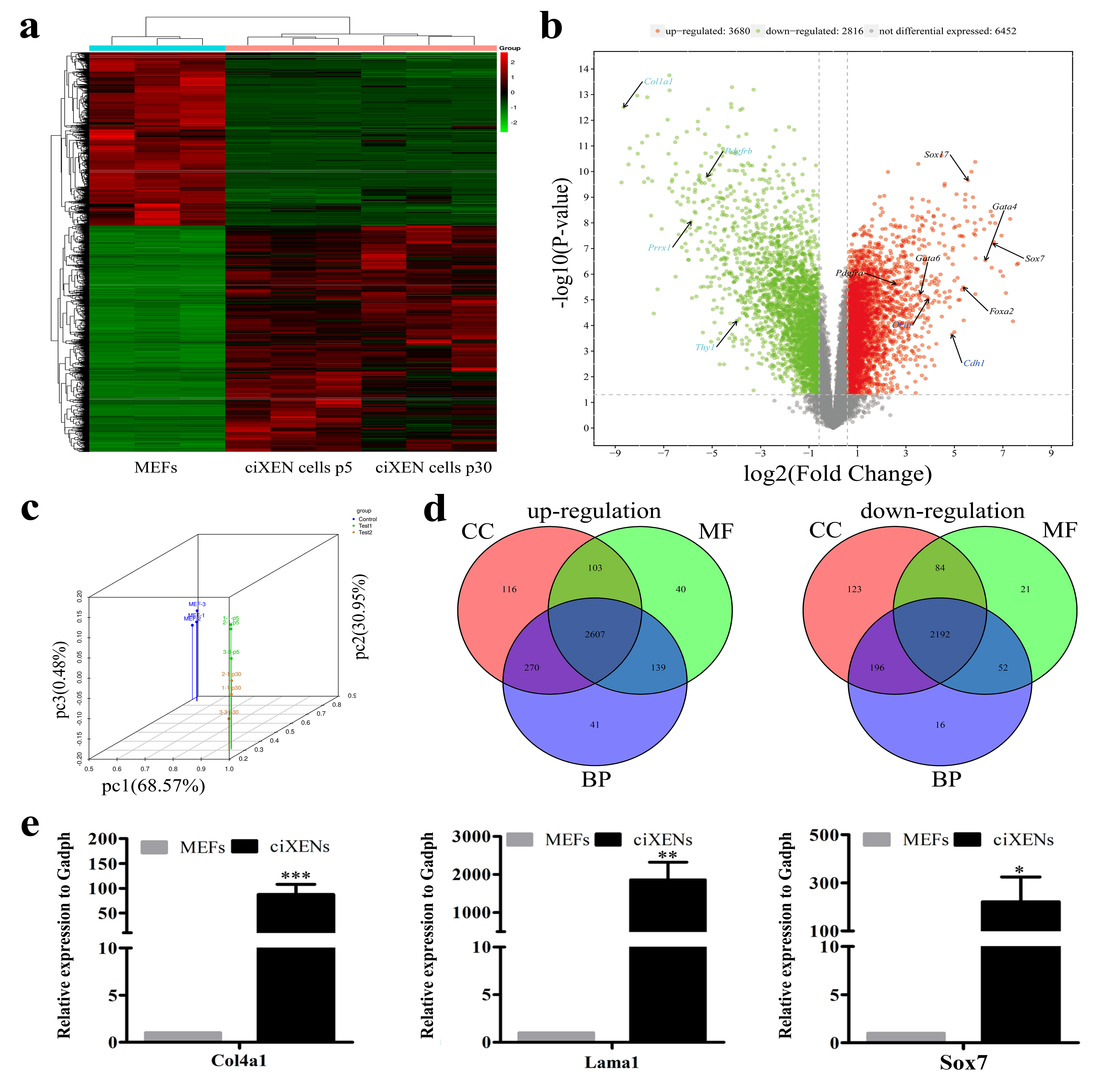

Supplement: Supplementary file 1 — Additional file 1 : Figure S1. Small molecules induced mouse fibroblasts to transform into ciXEN cells. Expression of Epcam (a) and Cxcr4 (b) and mesenchymal markers (c) during chemical induction as measured by qPCR. d Western blot analysis for the expression of E-cadherin and Vimentin during chemical induction. e Morphological changes of MNFs induced by chemicals and bFGF (bar, 100 μm). f Numbers of cell clones from different numbers of initial cells: 3w, 4w, and 5w. g Co-immunostaining for the expression of Sox17 and Foxa2 in MNF-derived clone (bar, 50 μm). * p < 0.05, ** p < 0.01, *** p < 0.001. Mean ± SEM, t-test and two-way ANOVA analysis, n ≥ 3. Figure S2. Characteristics of ciXEN cells at different passages. qPCR results for the expression of Epcam, Pdgfra, (a) and Cxcr4 (b) in ciXEN cells at p5. c The ultrastructure of MEFs and ciXEN cells. The thin arrow shows the endoplasmic reticulum (white) in MEFs and the cilium (black) in ciXEN cells; the thick arrow shows the mitochondria in ciXEN cells. (bar, 2 μm and 1 μm). d Cell morphologies of ciXEN cells at p5, p10, p20, p30 (bar, 100 μm). e Karyotype analysis of MEFs and ciXEN cells at p5, p15, and p30. mRNA levels of fibroblast genes (Prrx1, Pdgfrb, Col1a1, and Thy1), epithelial genes (Ocln and Cdh1) (f), XEN markers (Sox17, Foxa2, Gata4, and Gata6) (g), and pluripotent genes (Oct4, Sox2, and Nanog) (h). p15: passage 15; p20: passage 20. * p < 0.05, ** p < 0.01, *** p < 0.001. Mean ± SEM, t-test and two-way ANOVA analysis, n = 3. Figure S3. The metabolic patterns of ciXEN cells. a OCR of MEFs and ciXEN cells. b Total ATP of MEFs and ciXEN cells. Expression of Glut1 (c) and Pfk1, Ldha, and Hk2 (d) during chemical induction as measured by qPCR. e Numbers of cell clones under different treatment conditions: MEFs + VPACRFE and MEFs + VPACRFE + PS48. * p < 0.05, ** p < 0.01, *** p < 0.001. Mean ± SEM, t-test, n = 3. Figure S4. PCR/qPCR analysis for the expression of hepatic genes during the induction of iHeps. * p [file 13287_2020_1664_MOESM1_ESM.zip › updated figures/Fig.3.tif]

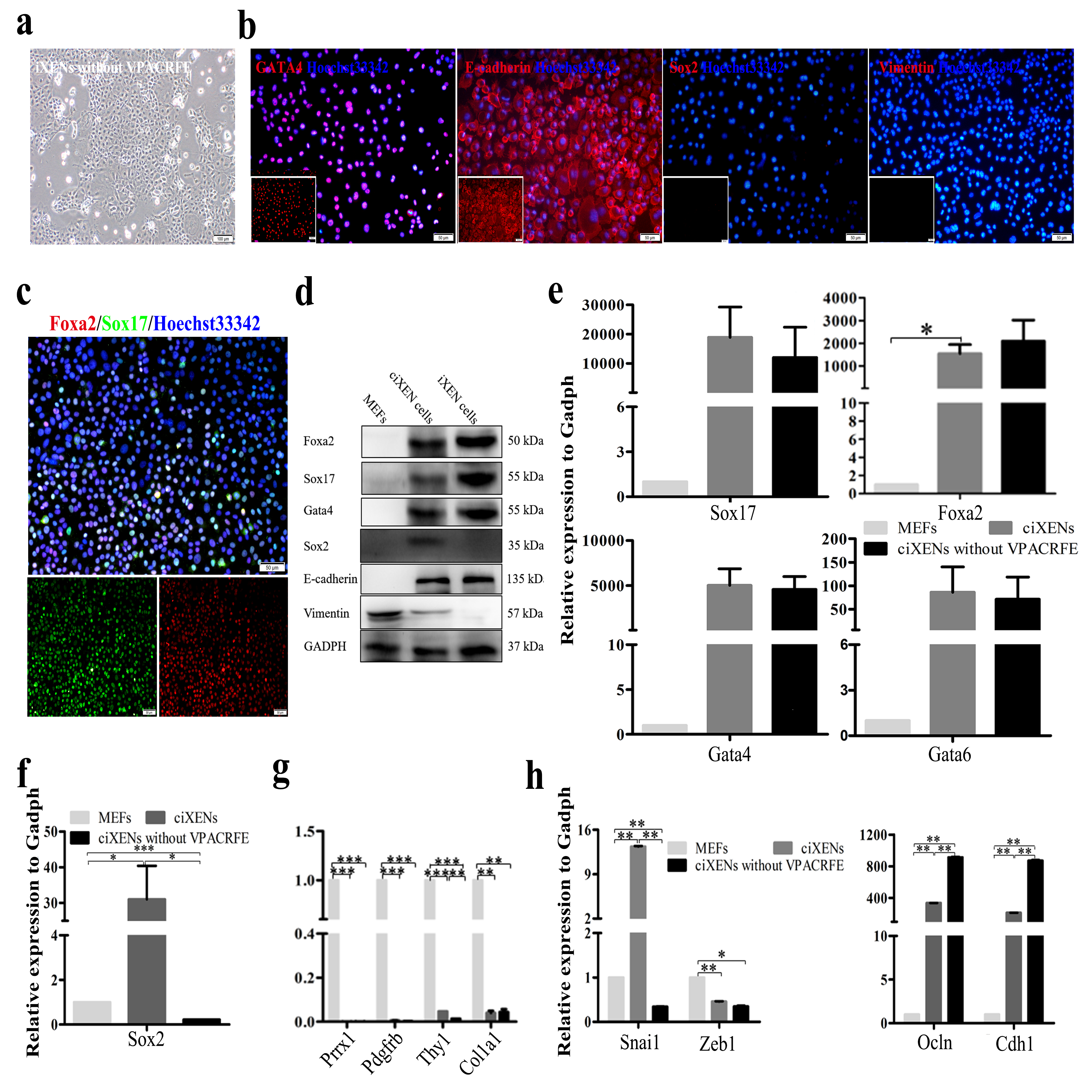

Supplement: Supplementary file 1 — Additional file 1 : Figure S1. Small molecules induced mouse fibroblasts to transform into ciXEN cells. Expression of Epcam (a) and Cxcr4 (b) and mesenchymal markers (c) during chemical induction as measured by qPCR. d Western blot analysis for the expression of E-cadherin and Vimentin during chemical induction. e Morphological changes of MNFs induced by chemicals and bFGF (bar, 100 μm). f Numbers of cell clones from different numbers of initial cells: 3w, 4w, and 5w. g Co-immunostaining for the expression of Sox17 and Foxa2 in MNF-derived clone (bar, 50 μm). * p < 0.05, ** p < 0.01, *** p < 0.001. Mean ± SEM, t-test and two-way ANOVA analysis, n ≥ 3. Figure S2. Characteristics of ciXEN cells at different passages. qPCR results for the expression of Epcam, Pdgfra, (a) and Cxcr4 (b) in ciXEN cells at p5. c The ultrastructure of MEFs and ciXEN cells. The thin arrow shows the endoplasmic reticulum (white) in MEFs and the cilium (black) in ciXEN cells; the thick arrow shows the mitochondria in ciXEN cells. (bar, 2 μm and 1 μm). d Cell morphologies of ciXEN cells at p5, p10, p20, p30 (bar, 100 μm). e Karyotype analysis of MEFs and ciXEN cells at p5, p15, and p30. mRNA levels of fibroblast genes (Prrx1, Pdgfrb, Col1a1, and Thy1), epithelial genes (Ocln and Cdh1) (f), XEN markers (Sox17, Foxa2, Gata4, and Gata6) (g), and pluripotent genes (Oct4, Sox2, and Nanog) (h). p15: passage 15; p20: passage 20. * p < 0.05, ** p < 0.01, *** p < 0.001. Mean ± SEM, t-test and two-way ANOVA analysis, n = 3. Figure S3. The metabolic patterns of ciXEN cells. a OCR of MEFs and ciXEN cells. b Total ATP of MEFs and ciXEN cells. Expression of Glut1 (c) and Pfk1, Ldha, and Hk2 (d) during chemical induction as measured by qPCR. e Numbers of cell clones under different treatment conditions: MEFs + VPACRFE and MEFs + VPACRFE + PS48. * p < 0.05, ** p < 0.01, *** p < 0.001. Mean ± SEM, t-test, n = 3. Figure S4. PCR/qPCR analysis for the expression of hepatic genes during the induction of iHeps. * p [file 13287_2020_1664_MOESM1_ESM.zip › updated figures/Fig.7.tif]
