# Supplementary material for: Characterisation of extraembryonic endoderm-like cells from mouse embryonic fibroblasts induced using chemicals alone
Source: Stem Cell Res Ther. 2020 Apr 16;11:157. doi: 10.1186/s13287-020-01664-0 (PMC7164364; doi:10.1186/s13287-020-01664-0)
Supplement: Supplementary file 2 — Additional file 2 : Table S1. Primers used for PCR/qPCR. Table S2. GO analysis of the top 10 upregulated CCs and MFs, and the top 10 downregulated CCs and MFs in ciXEN cells at passage 5 and passage 30 compared to those in MEFs. Table S3. Pathway analysis of the upregulated metabolic pathways in ciXEN cells at passage 5 compared to those in MEFs. [file 13287_2020_1664_MOESM2_ESM.zip › Table S1.docx]

**Table S1**

| Genes | Forward primer | Reverse primer |
| --- | --- | --- |
| Sox2 | CGGGAAGCGTGTACTTATCCTT | GCGGAGTGGAAACTTTTGTCC |
| Oct4 | CAGGGCTTTCATGTCCTGG | AGTTGGCGTGGAGACTTTGC |
| Nanog | AGTTATGGAGCGGAGCAGCAT | AGGCCTGGACCGCTCAGT |
| Gata6 | TGAGGTGGTCGCTTGTGTAG | ATGGCGTAGAAATGCTGAGG |
| Gata4 | GAGCTGGCCTGCGATGTCTGAGTG | AAACGGAAGCCCAAGAACCTGAAT |
| Sox17 | GTCAACGCCTTCCAAGACTTG | GTAAAGGTGAAAGGCGAGGTG |
| Sox7 | GAGCATGGTCACCCCCATC | AGGGCTAAAGAACCTAGAGGG |
| Foxa2 | CCTGGCTGCAGACACTTCCTAC | CAGGGCCTGAAAGCCATCTT |
| Hnf4a | CATCACCACCATCGTCAA | CCTCGTGTCACATCTTCTT |
| Alb | ATGTTACCAAGTGCTGTAGT | AATCTGCTTCTCCTTCTCTG |
| Afp | TTCCTGTCTCAGTCATTCTAAG | AGTCTCCTAAGGTCTGGTAG |
| Ttr | CTCACCACAGATGAGAAG | GGCTGAGTCTCTCAATTC |
| Prrx1 | AGTCACCGGGACTGACCA | TCCGCTGCTTTCTCTTCTTC |
| Col1a1 | CCCTGCCTGCTTCGTGTAAA | TCGTCTGTTTCCAGGGTTGG |
| Pdgfrβ | CAGGACCTCTGGCTGAAGCA | TCTGGGAGGCAGAAGGGAGAT |
| Col4a1 | AAAGGGAGAAAGAGGCTTGC | CTCCAGCATCACCCTTTTGT |
| Lama1 | CTCGGTGCTCTGGGTCAC | ACATCTCAGGCCCCTTCTCT |
| Pdgfra | AGTGGCTACATCATCCCCCT | CCGAAGTCTGTGAGCTGTGT |
| Cxcr4 | TCCAACAAGGAACCCTGCTTC | TTGCCGACTATGCCAGTCAAG |
| Vimentin | GCCTGCAGGATGAGATCCAA | AAAAGGTTGGCAGAGGCAGA |
| Snai1 | CTTGTGTCTGCACGACCTGT | AGTGGGAGCAGGAGAATGG |
| Zeb1 | CCAGGTGTAAGCGCAGAAAG | TCATCGGAATCTGAATTTGC |
| Twist2 | GTCTCAGCTACGCCTTCTCC | GCCTGAGATGTGCAGGTG |
| Thy1 | AACTCTTGGCACCATGAACC | GTCAGGCTGGTCACCTTCTG |
| Cyp2a5 | GCACTTCCTAGATGACAAGGGACA | CAGGCTCAACGGGACAAGAA |
| Cyp3a11 | TACTGTGATGGAGATGGAATAC | GGTGAAGAGCATAAGATGGA |
| Hnf6a | AAATAAGCGTCCGTCCAAAGAA | GACGATGAACTGCCTGAGTTG |
| Cdh1 | CTCAAGCTCGCGGATAACCA | AATCCTGCTGCCACGATTC |
| Col1a1 | CCCTGCCTGCTTCGTGTAAA | TCGTCTGTTTCCAGGGTTGG |
| Ocln | TCGCACATCAAGAGGATGGTG | GCCTCTGGAGAGAATTGCAGAGA |
| Epcam | AACACAAGACGACGTGGACA | ACTCGGGTGCCTTTTCATCA |
| Apoa1 | GTGGCTCTGGTCTTCCTGAC | ACGGTTGAACCCAGAGTGTC |
| Hk2 | AGACGGTGAGAGATCTGGCT | GGCCACAGCAGTGATGAGAG |
| Ldha | TCAGGCGGCTACACGTACA | GGAGATCCATCATCTCGCCC |
| Pfk1 | TGCCAATGCTCCAGACTCAG | AGATTCAGCCACCACTGCTC |
| Glut1 | GATCCCAGCAGCAAGAAGGT | GAGAGACCAAAGCGTGGTGA |
| Gadph | CATGTTCCAGTATGACTCCACTC | GGCCTCACCCCATTTGATGT |
