# Supplementary material for: Characterisation of extraembryonic endoderm-like cells from mouse embryonic fibroblasts induced using chemicals alone
Source: Stem Cell Res Ther. 2020 Apr 16;11:157. doi: 10.1186/s13287-020-01664-0 (PMC7164364; doi:10.1186/s13287-020-01664-0)
Supplement: Supplementary file 2 — Additional file 2 : Table S1. Primers used for PCR/qPCR. Table S2. GO analysis of the top 10 upregulated CCs and MFs, and the top 10 downregulated CCs and MFs in ciXEN cells at passage 5 and passage 30 compared to those in MEFs. Table S3. Pathway analysis of the upregulated metabolic pathways in ciXEN cells at passage 5 compared to those in MEFs. [file 13287_2020_1664_MOESM2_ESM.zip › Table S2.docx]

**Table S2**

| ID | Term | Ontology | Count | P_value | FDR | Regulation |
| --- | --- | --- | --- | --- | --- | --- |
|  |  |  |  |  |  |  |
| GO:0005622 | intracellular | Cellular Component | 2912 | 1.2E-248 | 1.4E-245 | Up |
| GO:0043229 | intracellular_organelle | Cellular Component | 2629 | 4.1E-219 | 2.3E-216 | Up |
| GO:0043231 | intracellular_membrane-bounded_organelle | Cellular Component | 2353 | 2.3E-202 | 8.6E-200 | Up |
| GO:0043227 | membrane-bounded_organelle | Cellular Component | 2443 | 1.3E-194 | 3.9E-192 | Up |
| GO:0005623 | cell | Cellular Component | 3079 | 1.5E-179 | 3.5E-177 | Up |
| GO:0005634 | nucleus | Cellular Component | 1653 | 5.1E-139 | 9.8E-137 | Up |
| GO:0005737 | cytoplasm | Cellular Component | 2218 | 1.3E-107 | 2.2E-105 | Up |
| GO:0031981 | nuclear_lumen | Cellular Component | 962 | 4.33E-96 | 6.2E-94 | Up |
| GO:0032991 | protein-containing_complex | Cellular Component | 1252 | 6.17E-71 | 7.85E-69 | Up |
| GO:0005654 | nucleoplasm | Cellular Component | 724 | 1.14E-70 | 1.3E-68 | Up |
| GO:0097159 | organic_cyclic_compound_binding | Molecular Function | 1223 | 1.76E-86 | 3.92E-83 | Up |
| GO:1901363 | heterocyclic_compound_binding | Molecular Function | 1205 | 4.13E-86 | 4.59E-83 | Up |
| GO:0003676 | nucleic_acid_binding | Molecular Function | 823 | 1.3E-74 | 9.63E-72 | Up |
| GO:0003824 | catalytic_activity | Molecular Function | 1251 | 1.09E-57 | 6.04E-55 | Up |
| GO:0003677 | DNA_binding | Molecular Function | 558 | 1E-51 | 4.47E-49 | Up |
| GO:0033218 | amide_binding | Molecular Function | 1683 | 5.19E-38 | 1.93E-35 | Up |
| GO:0005515 | protein_binding | Molecular Function | 1659 | 3.49E-37 | 1.11E-34 | Up |
| GO:0042277 | peptide_binding | Molecular Function | 1671 | 9.16E-37 | 2.55E-34 | Up |
| GO:0043167 | ion_binding | Molecular Function | 1091 | 2.15E-35 | 5.33E-33 | Up |
| GO:0036094 | small_molecule_binding | Molecular Function | 556 | 4.92E-31 | 1.1E-28 | Up |
| GO:0005623 | cell | Cellular Component | 2295 | 1.14E-93 | 1.06E-90 | Down |
| GO:0005737 | cytoplasm | Cellular Component | 1709 | 3.30E-79 | 1.53E-76 | Down |
| GO:0005622 | intracellular | Cellular Component | 1998 | 4.54E-71 | 1.40E-68 | Down |
| GO:0071944 | cell_periphery | Cellular Component | 952 | 9.63E-58 | 2.23E-55 | Down |
| GO:0031012 | extracellular_matrix | Cellular Component | 143 | 2.35E-54 | 4.34E-52 | Down |
| GO:0005886 | plasma_membrane | Cellular Component | 922 | 4.37E-54 | 6.74E-52 | Down |
| GO:0005576 | extracellular_region | Cellular Component | 516 | 3.00E-47 | 3.97E-45 | Down |
| GO:0043229 | intracellular_organelle | Cellular Component | 1716 | 9.17E-45 | 1.06E-42 | Down |
| GO:0042995 | cell_projection | Cellular Component | 497 | 1.80E-43 | 1.85E-41 | Down |
| GO:0016020 | membrane | Cellular Component | 1411 | 2.50E-42 | 2.32E-40 | Down |
| GO:0005515 | protein_binding | Molecular Function | 1667 | 5.98E-153 | 1.14E-149 | Down |
| GO:0042277 | peptide_binding | Molecular Function | 1677 | 1.92E-152 | 1.83E-149 | Down |
| GO:0033218 | amide_binding | Molecular Function | 1680 | 1.27E-151 | 8.09E-149 | Down |
| GO:0043167 | ion_binding | Molecular Function | 983 | 5.14E-62 | 2.45E-59 | Down |
| GO:0046872 | metal_ion_binding | Molecular Function | 639 | 1.84E-39 | 7.00E-37 | Down |
| GO:0043169 | cation_binding | Molecular Function | 649 | 3.36E-38 | 1.07E-35 | Down |
| GO:0008092 | cytoskeletal_protein_binding | Molecular Function | 248 | 2.31E-37 | 6.30E-35 | Down |
| GO:0042802 | identical_protein_binding | Molecular Function | 391 | 2.37E-35 | 5.66E-33 | Down |
| GO:0005102 | signaling_receptor_binding | Molecular Function | 381 | 5.78E-35 | 1.23E-32 | Down |
| GO:0019899 | enzyme_binding | Molecular Function | 451 | 1.86E-32 | 3.55E-30 | Down |
