# Supplementary material for: Characterisation of extraembryonic endoderm-like cells from mouse embryonic fibroblasts induced using chemicals alone
Source: Stem Cell Res Ther. 2020 Apr 16;11:157. doi: 10.1186/s13287-020-01664-0 (PMC7164364; doi:10.1186/s13287-020-01664-0)
Supplement: Supplementary file 2 — Additional file 2 : Table S1. Primers used for PCR/qPCR. Table S2. GO analysis of the top 10 upregulated CCs and MFs, and the top 10 downregulated CCs and MFs in ciXEN cells at passage 5 and passage 30 compared to those in MEFs. Table S3. Pathway analysis of the upregulated metabolic pathways in ciXEN cells at passage 5 compared to those in MEFs. [file 13287_2020_1664_MOESM2_ESM.zip › Table S3.docx]

**Table S3**

| ID | Term | Count | p value | FDR |
| --- | --- | --- | --- | --- |
| mmu00480 | Glutathione metabolism | 25 | 1.85345E-05 | 0.000494617 |
| mmu00240 | Pyrimidine metabolism | 30 | 0.001611521 | 0.02628956 |
| mmu00270 | Cysteine and methionine metabolism | 16 | 0.007025541 | 0.086554661 |
| mmu00230 | Purine metabolism | 44 | 0.009899595 | 0.105140525 |
| mmu01200 | Carbon metabolism | 31 | 0.013748758 | 0.136600561 |
| mmu00051 | Fructose and mannose metabolism | 12 | 0.014576959 | 0.140303234 |
| mmu00020 | Citrate cycle (TCA cycle) | 11 | 0.018614516 | 0.163807741 |
| mmu00052 | Galactose metabolism | 11 | 0.018614516 | 0.163807741 |
| mmu00630 | Glyoxylate and dicarboxylate metabolism | 10 | 0.02383291 | 0.198392874 |
| mmu00640 | Propanoate metabolism | 10 | 0.037680555 | 0.267333094 |
| mmu00740 | Riboflavin metabolism | 4 | 0.03842795 | 0.267333094 |
| mmu00670 | One carbon pool by folate | 7 | 0.039058407 | 0.267333094 |
| mmu00030 | Pentose phosphate pathway | 10 | 0.046346937 | 0.31032297 |
| mmu00561 | Glycerolipid metabolism | 16 | 0.049842483 | 0.319822601 |
